# Development of insomnia in patients with stroke: A systematic review and meta-analysis

**DOI:** 10.1371/journal.pone.0297941

**Published:** 2024-04-10

**Authors:** Junwei Yang, Aitao Lin, Qingjing Tan, Weihua Dou, Jinyu Wu, Yang Zhang, Haohai Lin, Baoping Wei, Jiemin Huang, Juanjuan Xie

**Affiliations:** 1 The First Affiliated Hospital of Guangxi University of Chinese Medicine, Nanning, Guangxi, 530023, China; 2 Guangxi University of Traditional Chinese Medicine, Nanning, Guangxi, 530001, China; University of Rijeka Faculty of Medicine: Sveuciliste u Rijeci Medicinski fakultet, CROATIA

## Abstract

**Background and aim:**

Stroke is a serious threat to human life and health, and post-stroke insomnia is one of the common complications severely impairing patients’ quality of life and delaying recovery. Early understanding of the relationship between stroke and post-stroke insomnia can provide clinical evidence for preventing and treating post-stroke insomnia. This study was to investigate the prevalence of insomnia in patients with stroke.

**Methods:**

The Web of Science, PubMed, Embase, and Cochrane Library databases were used to obtain the eligible studies until June 2023. The quality assessment was performed to extract valid data for meta-analysis.

The prevalence rates were used a random-efect. *I*^*2*^ statistics were used to assess the heterogeneity of the studies.

**Results:**

1. Twenty-six studies met the inclusion criteria for meta-analysis, with 1,193,659 participants, of which 497,124 were patients with stroke.
2. The meta-analysis indicated that 150,181 patients with stroke developed insomnia during follow-up [46.98%, 95% confidence interval (CI): 36.91–57.18] and 1806 patients with ischemic stroke (IS) or transient ischemic attack (TIA) developed insomnia (47.21%, 95% CI: 34.26–60.36). Notably, 41.51% of patients with the prevalence of nonclassified stroke developed insomnia (95% CI: 28.86–54.75). The incidence of insomnia was significantly higher in patients with acute strokes than in patients with nonacute strokes (59.16% vs 44.07%, *P* < 0.0001).
3. Similarly, the incidence of insomnia was significantly higher in the patients with stroke at a mean age of ≥65 than patients with stroke at a mean age of <65 years (47.18% vs 40.50%, *P* < 0.05). Fifteen studies reported the follow-up time. The incidence of insomnia was significantly higher in the follow-up for ≥3 years than follow-up for <3 years (58.06% vs 43.83%, *P* < 0.05). Twenty-one studies used the Insomnia Assessment Diagnostic Tool, and the rate of insomnia in patients with stroke was 49.31% (95% CI: 38.59–60.06). Five studies used self-reporting, that the rate of insomnia in patients with stroke was 37.58% (95% CI: 13.44–65.63).

**Conclusions:**

Stroke may be a predisposing factor for insomnia. Insomnia is more likely to occur in acute-phase stroke, and the prevalence of insomnia increases with patient age and follow-up time. Further, the rate of insomnia is higher in patients with stroke who use the Insomnia Assessment Diagnostic Tool.

## 1 Introduction

Stroke is the second most morbid and deadly disease globally, which is characterized by high morbidity, disability, mortality, and recurrence. It substantially threatens human life, health, and quality of life [1,2]. Previous study revealed that neuropsychiatric disorders frequently affect stroke survivors, such as insomnia, depression, or anxiety and so on [3]. Similarly, One third of stroke patients met the diagnostic criteria of insomnia, and patients may experience difficulty falling asleep, difficulty with sleep persistence, and early awakening [4].

Insomnia is the most common sleep disorder prevalent in people of all ages. In severe cases, it can affect daytime work and life, and even cause emotional disorders [5]. The incidence of insomnia increases with the increase in social pressure [6]. Study showed that the incidence of insomnia in stroke patients is higher than the normal healthy population, and some patients with insomnia may be more prone to stroke risk [7]. As increasing studies showed that insomnia has a bidirectional relationship with stroke, which may be an independent risk factor for stroke. Further, stroke may also be a predisposing factor for insomnia [8]. Therefore, it is essential to understand the relationship between stroke and post-stroke insomnia in an early stage to provide a clinical basis for the early prevention and treatment of post-stroke insomnia. The study aimed to investigate the prevalence of insomnia in patients with stroke.

## 2 Research design and method

The study was conducted and designed in strict accordance with the Preferred Reporting Items for Systematic Reviews and Meta-Analyses (PRISMA) guidelines [9,10].

### 2.1 Data source and selection process

Literature related to the occurrence of developmental insomnia in stroke patients was collected through PubMed, The Cochrane Library, Web of Science, and Embase databases until June 2023.

### 2.2 Search strategy

**Fig 1:**
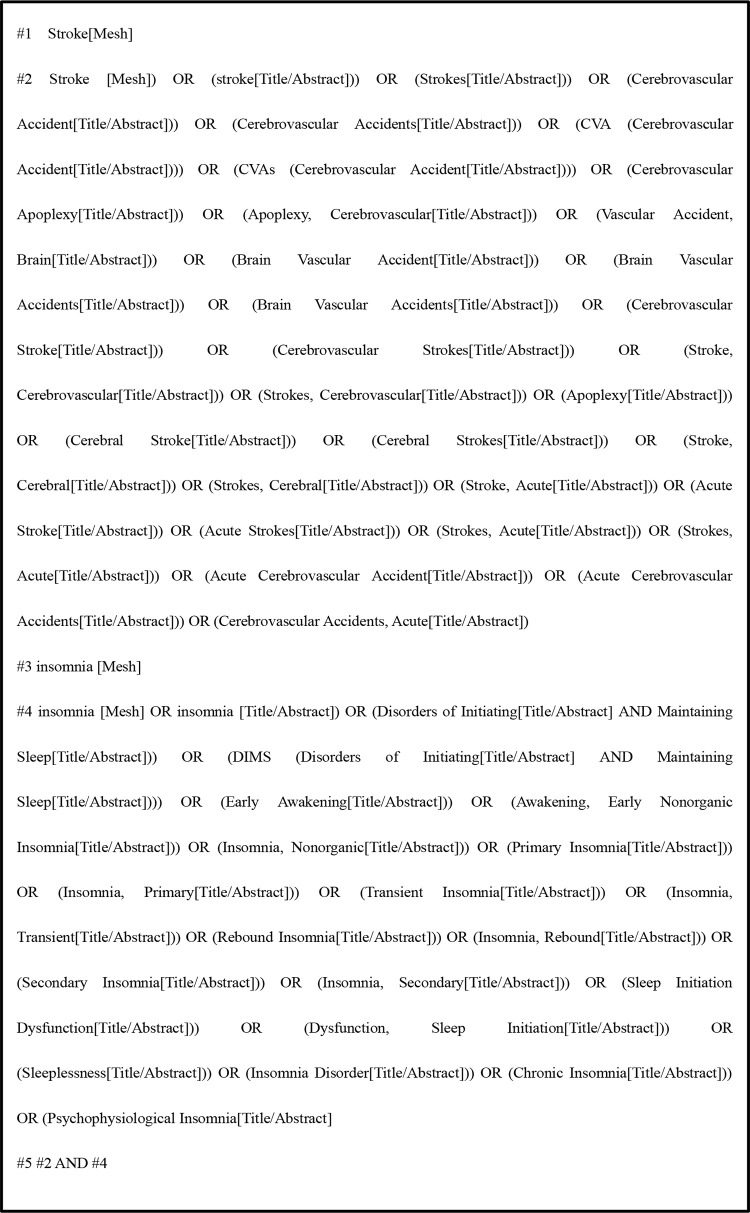
Search strategy of PubMed.

We searched the related literature by the subject terms, such as “Stroke”, “Cerebrovascular Accident”, “Insomnia”, “Insomnia Disorder”, etc. The following search strategy for the PubMed database (Fig 1).

### 2.3 Eligibility criteria and study selection

In the study, we included the cohort studies and cross-sectional studies about stroke patients who developed insomnia in English language. Stroke patients met the diagnostic criteria of the Essentials of Diagnosis of Various Cerebrovascular Diseases [11]. Insomnia patients were diagnosed through recognized assessment tools such as the Pittsburgh Sleep Quality Index (PSQI), Hamilton Depression Scale (HDS), or self-reported symptoms of insomnia and met the diagnostic criteria of the American Academy of Sleep in 2014 [12]. We excluded the duplicate records, case reports, reviews and so on.

### 2.4 Exclusion criteria

We excluded the duplicate literature, case reports, reviews and the the literature with incomplete data indicators, or the information was not available.

### 2.5 Data extraction

#### 2.5.1 Literature screening and information extraction

LAT and TQJ screened the included literatures. The extracted information mainly included the basic information of the literatures: first author name, the time of publication, sample size, the country, the follow-up time and the number of positive cases. In case of disagreement between two researchers in the literature screening or data extraction process, the decision was submitted to the third researcher (YJW).

#### 2.5.2 Literature quality assessment

The methodological quality of the included studies was assessed using the Critical Appraisal Tool for Prevalence Studies [13,14]. Any disagreements by the researchers were submitted to a third researcher (YJW).

### 2.6 Statistical analysis

In the study, we used systematic Meta-Analysis software version 3 to calculate the statistical analyse [15]. The fixed effects model was used in *P*≥0.10 and *I*^*2*^≤50%, and random-effects model was used in *P*<0.10 and/or *I*^*2*^>50%, which was necessary to find the source of heterogeneity and perform subgroup analysis or sensitivity analysis [16–18].

## 3 Results

### 3.1 PROSPERO registration

Registration number: CRD42023452419.

### 3.2 Literature search results

**Fig 2:**
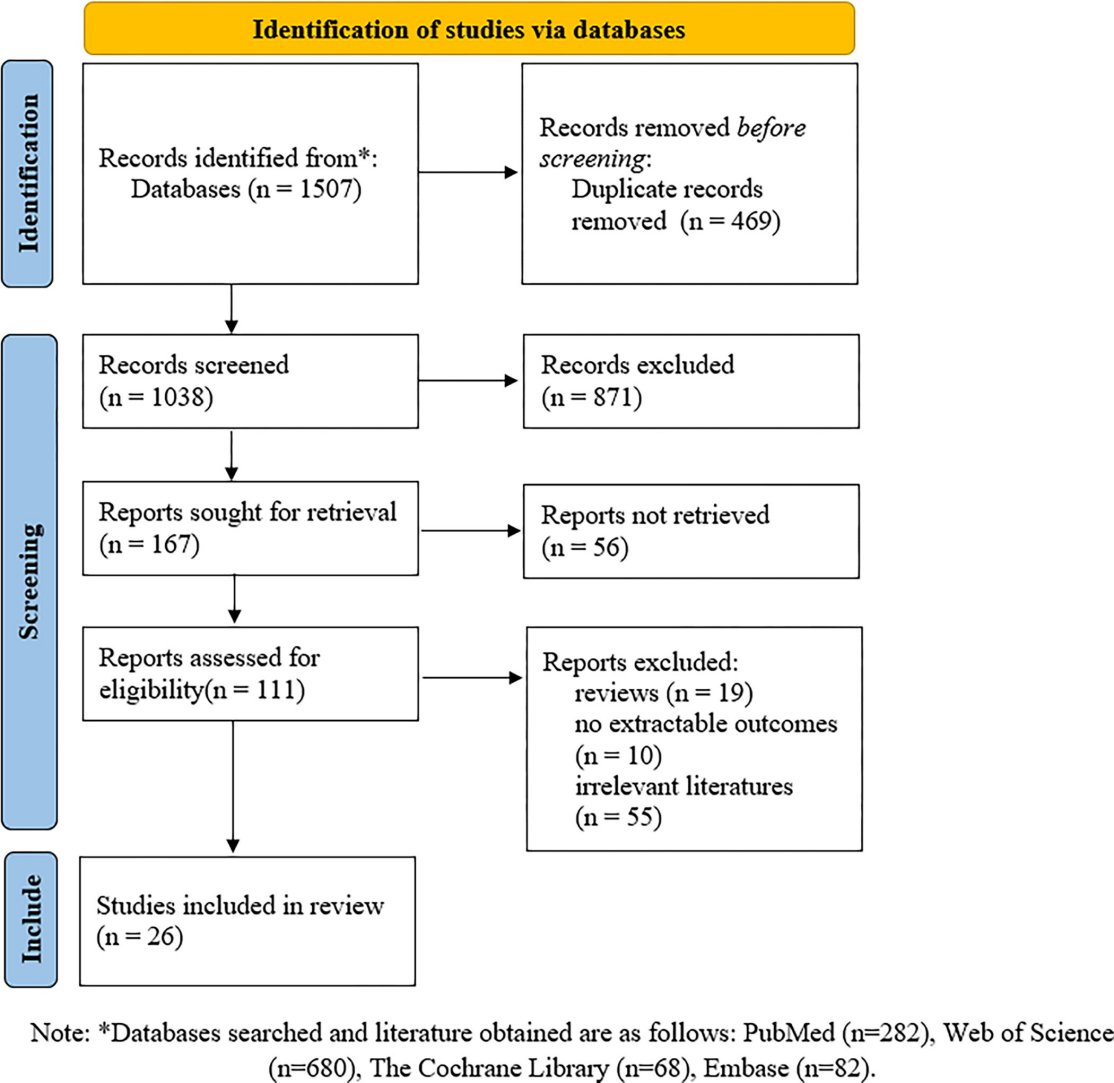
Literature screening process and results.

We got 1507 literatures from databases, of which 469 were duplicates and hence excluded. Further, we excluded 927 studies by the exclusion criteria. Overall, 111 studies were retained for the full-text evaluation, and finally 26 studies were included in the meta-analysis (Fig 2) [7,19–43].

### 3.4 Basic characteristics of the included studies

The 26 included studies (13 prospective cohort studies, 10 cross-sectional studies, 2 retrospective studies, and 1 multicenter observational study) were published between 2002 and 2023. Overall, the 26 studies included had 1,193,659 participants, of which 497,124 were patients with stroke. The details are shown in Table 1.

**Table 1:** Study characteristics.

Study details								Sample characteristics (stroke sample)		
First author/Date	Country	Years	Total N (%, n of stroke)	Design	Stroke/n	Insomnia/n	Followup period	Mean Age, years (SD)	Stroke type	Gender % male
Simone B. Duss et al 2023[19]	Bern	2015.11–2016.7	437 (100%)	Prospective cohort study	437	168	2 yrs	65.1 ± 13.0	IS、TIA	63.8%
Won-Hyoung Kim et al 2017[20]	Korean	2014.3–2015.12	214 (100%)	Cohort study	214	128	1 mo	NR	IS、Hemorrhagic stroke	NR
Hye-Mi Moon et al 2019[21]	Korean	2010–2012, 2013	504 (100%)	Cross-sectional Population based survey	504	123	NR	64.4 ± 0.7	stroke	55.7%
A. Leppävuori et al 2002[7]	NR	NR	277 (100%)	Cross-sectional interview	277	157	3 mo	70.7 ± 7.5	IS	50.9%
Ruo-lin Zhu et al 2022[22]	China	2020.1–2021.5	94 (100%)	Cross-sectional survey	94	59	16 mo	NR	IS	70.2%
A. Katharina Helbig et al 2015[23]	Germany	NR	15746 (5.8%, n = 917)	Cross-sectional survey	917	769	14 yrs (md)	NR	stroke	62.16%
Azizi A Seixas et al 2019[24]	USA	2000–2015	1108043 (43.9%, n = 486619)	Cross-sectional Population based survey	486619	145207	NR	47.5 ± 14.15	stroke	47.3%
Biljana Kojic et al 2021[25]	Tuzla	NR	110 (100%)	Prospective study	100	100	NR	65.13 ± 9.27	Stroke	59%
Chien-Yi Hsu et al 2015[26]	Taiwan,China	NR	44080 (2.38%, n = 1049)	Cross-sectional cohort	1049	743	10 yrs	NR	Stroke	NR
Faizul Hasan et al 2023[27]	Taiwan,China	2004.1–2017.9	1775 (100%)	Retrospective Cohort Study	1775	146	NR	67.6 ± 14.91	Stroke	58.6%
Gul M C et al 2016[28]	Turkey	NR	81 (100%)	Retrospective study	81	30	5 yrs	66.5 ± 10.3	IS	50.6%
Hui-Ju Tsai et al 2022[29]	Taiwan,China	2020.7–2021.10	195 (100%)	Prospective study	195	58	15 mo	64.1 ± 8.9	IS	59.5%
Ipek Sonmez et al 2019[30]	Famagusta	2016.1–2017.2	55 (100%)	Cross-sectional observational study	55	32	NR	69 ± 11	Stroke	NR
Jinil Kim et al 2015[31]	China	2013.10–2014.6	80 (100%)	Prospective study	80	57	NR	63.8 ± 13.6	IS、Hemorrhagic stroke	67.5%
Keun T K et al.2017[32]	Korean	NR	241 (100%)	Prospective study	241	108	NR	64.2 ± 11.9	AIS	60.6%
Kyung-Lim Joa et al 2017[33]	Korean	NR	208 (100%)	Multicenter-observational and correlation study	208	56	NR	61.53 ± 12.58	Stroke	54%
Li-Jun Li et al 2018[34]	China	2008.4–2010.4	1062 (100%)	Prospective Cohort Study	1062	489	6 yrs	60.47 ± 11.57	Stroke	65.7%
M. Sieminski et al 2009[35]	Poland	1995–2005	90 (100%)	Prospective study	90	65	NR	66.5 ± 12.8	IS	46.7%
Mayura T I et al 2019[36]	Australia	2016.8–2018.1	104 (100%)	prospective cohort study	104	31	17 mo	76 ± 7	Stroke	52.9%
Min-Y K et al 2018[37]	Korean	2010–2014	17601 (2%, n = 360)	Cross-sectional survey study	360	170	4 yrs	NR	Stroke	NR
Nick Glozier et al 2017[38]	Australia	2008–2010	368 (100%)	Prospective cohort study	368	124	1 year	NR	IS、Hemorrhagic stroke	68.2%
Wai-Kwong Tang et al 2015[39]	Hong Kong, China	2008.6–2011.9	336 (100%)	Cross-sectional survey study	336	149	3 mo	66.1 ± 10.2	Acute stroke	60.4%
Won-Hyoung Kim et al 2019[40]	Korean	2016.7–2018.8	112 (100%)	Cohort study	112	40	NR	NR	Stroke	54.5%
Xiao-Wei Fan et al 2022[41]	China	2015.8–2018.3	1619 (100%)	Prospective study	1619	1137	3 mo	60.8 ± 10.7	AIS or TIA	72.5%
Yitao He et al 2019[42]	China	2016.1–2018.6	152 (100%)	Prospective study	152	24	3 mo	65.25 ± 13.56	AIS	67.76%
Alia H. Mansour et al 2020[43]	Egypt	2015.1–2015.12	75 (100%)	Cross-sectional prospective study	75	11	NR	59.3 ± 5.34	Stroke	45.3%

### 3.5 Quality of included studies

Table 2 shows the quality assessment of the included studies. 69.23% (eighteen studies) studies determined the prevalence of insomnia in stroke patients in a sufficient sample size. To assess insomnia, most studies used standardized instruments or validated diagnostic criteria (80.77%). The details are shown in Table 2.

**Table 2:** Quality of included studies.

Study	Response									
	Q1	Q2	Q3	Q4	Q5	Q6	Q7	Q8	Q9	Q10
Simone B. Duss et al 2023[19]	Y	Y	Y	Y	Y	Y	N	Y	Y	Y
Won-Hyoung Kim et al 2017[20]	Y	Y	Y	Y	Y	Y	Y	Y	Y	Y
Hye-Mi Moon et al 2019[21]	Y	Y	Y	Y	Y	N	Y	Y	Y	Y
A. Leppävuori et al 2002[7]	Y	U	Y	Y	Y	Y	Y	Y	Y	Y
Ruo-lin Zhu et al 2022[22]	Y	Y	N	Y	Y	Y	Y	Y	Y	Y
K.H. et al 2015[23]	Y	Y	Y	Y	Y	U	U	Y	Y	Y
Azizi A Seixas et al 2019[24]	Y	Y	Y	Y	Y	N	Y	Y	Y	Y
Biljana Kojic et al 2021[25]	Y	U	Y	Y	Y	Y	Y	Y	Y	Y
Chien-Yi Hsu et al 2015[26]	Y	Y	Y	Y	Y	Y	Y	Y	Y	Y
Faizul Hasan et al 2023[27]	Y	Y	Y	Y	Y	N	Y	Y	Y	Y
Gul M C et al 2016[28]	Y	U	N	Y	Y	Y	Y	Y	Y	Y
Hui-Ju Tsai et al 2022[29]	Y	Y	Y	Y	Y	Y	Y	Y	Y	Y
Ipek Sonmez et al 2019[30]	Y	Y	Y	Y	Y	Y	U	Y	Y	Y
Jinil Kim et al 2015[31]	Y	Y	N	Y	Y	Y	U	Y	Y	Y
Keun T K et al.2017[32]	Y	Y	Y	Y	Y	Y	Y	Y	Y	Y
Kyung-Lim Joa et al 2017[33]	Y	Y	Y	Y	Y	Y	Y	Y	Y	Y
Li-Jun Li et al 2018[34]	Y	U	Y	Y	Y	Y	Y	Y	Y	Y
M. Sieminski et al 2009[35]	Y	U	N	U	Y	Y	Y	Y	Y	Y
Mayura T I et al 2019[36]	Y	U	N	Y	Y	Y	U	Y	Y	Y
Min-Y K et al 2018[37]	Y	Y	Y	Y	Y	N	N	Y	Y	Y
Nick Glozier et al 2017[38]	Y	Y	Y	Y	Y	Y	N	Y	Y	Y
Wai-Kwong Tang et al 2015[39]	Y	U	Y	Y	Y	Y	Y	Y	Y	Y
Won-Hyoung Kim et al 2019[40]	Y	U	N	Y	Y	Y	Y	Y	Y	Y
Xiao-Wei Fan et al 2022[41]	Y	Y	Y	Y	Y	Y	Y	Y	Y	Y
Yitao He et al 2019[42]	Y	Y	N	Y	Y	Y	Y	Y	Y	Y
Alia H. Mansour et al 2020[43]	Y	Y	N	Y	Y	Y	Y	Y	Y	Y

Keys

Q1-Q10 represents questions used to assess the quality of included studies, which are listed below.

Q1. Sample frame appropriate to address the target population.

Q2. Appropriate sampling of study participants.

Q3. Adequate sample size.

Q4. Study subjects and setting described in detail.

Q5. Data analysis conducted with sufficient coverage of the identified sample.

Q6. Valid methods used for the identification of insomnia or insomnia symptoms.

Q7. Valid methods used for the identification of stroke.

Q8. Condition measured in a standard, reliable way for all participants.

Q9. Appropriate statistical analysis.

Q10. Adequate response rate, if not, was low response rate managed appropriately.

### 3.6 Meta-analysis

**Fig 3:**
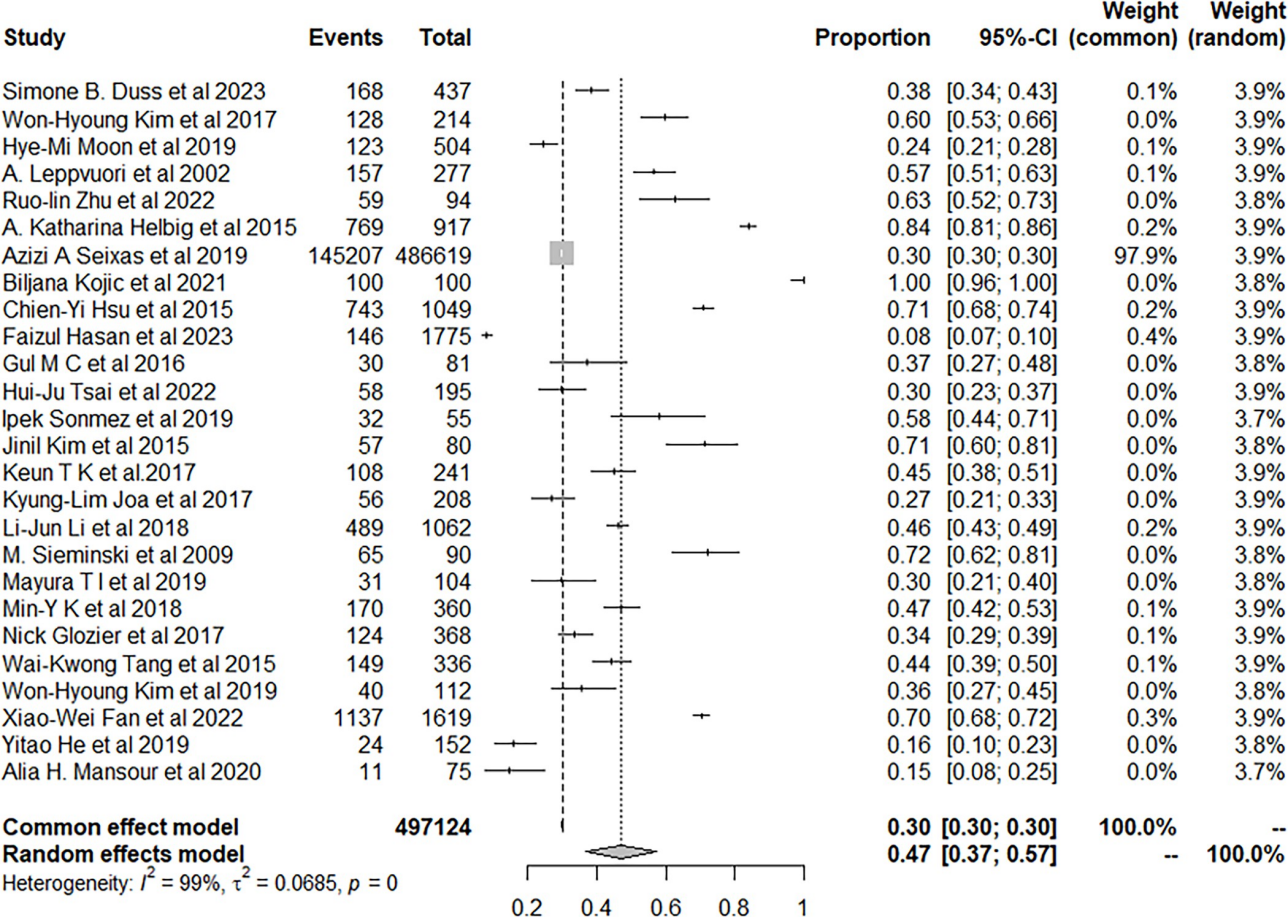
Forest plot of the meta-analysis of prevalence of insomnia among stroke patients.

We used the random-effects model to pool prevalence of insomnia in patients with stroke. 150,181 patients with stroke developed insomnia during the follow-up and the pool prevalence was 46.98% (95% CI: 36.91–57.18) (Fig 3).

**Fig 4:**
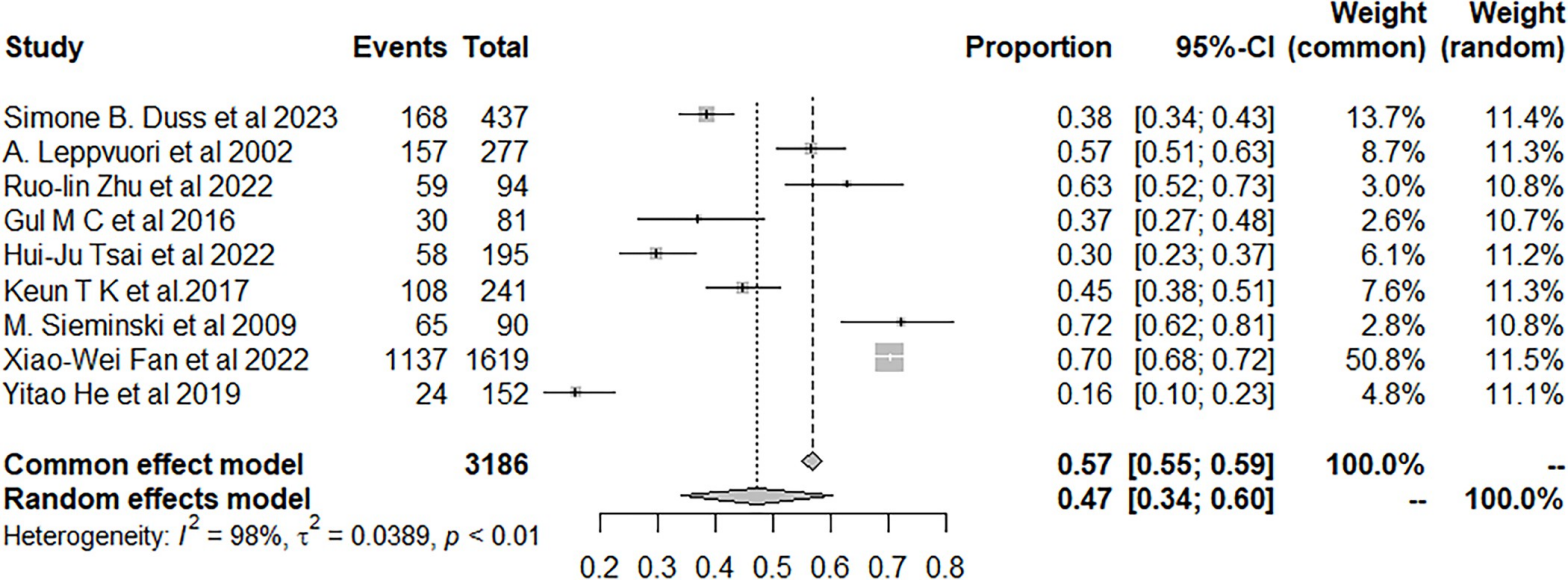
Forest plot of the meta-analysis of prevalence of insomnia among IS or TIA patients.

Moreover, nine studies examined the occurrence of insomnia in patients with IS or TIA. The result showed that the prevalence of insomnia among patients with IS or TIA was 47.21% (95% CI: 34.26–60.36) (Fig 4).

**Fig 5:**
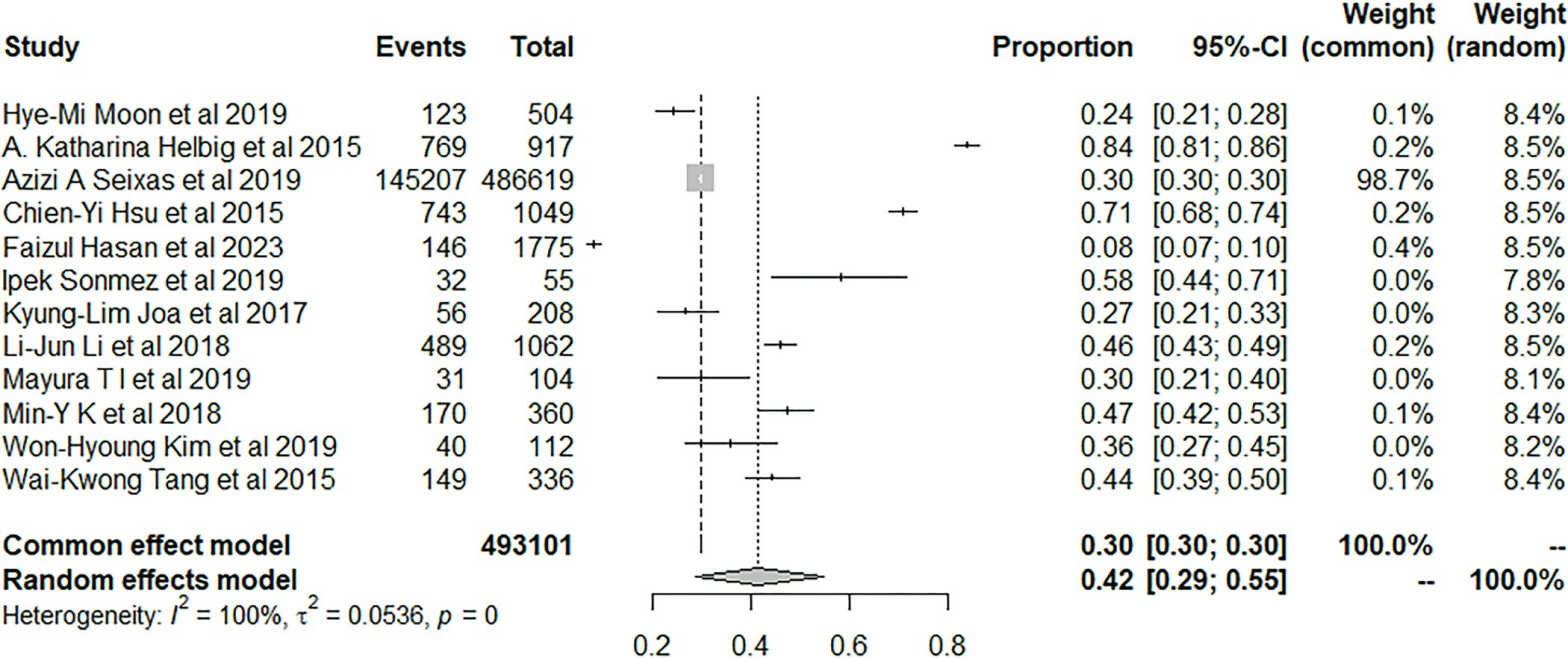
Forest plot of the meta-analysis of prevalence of insomnia among nonclassified stroke patients.

Four studies explicitly examined the prevalence of insomnia among IS or hemorrhage patients and the prevalence was 44.09% (95% CI: 19.84–69.92), while twelve studies did not specify the type of stroke (Fig 5).

**Fig 6:**
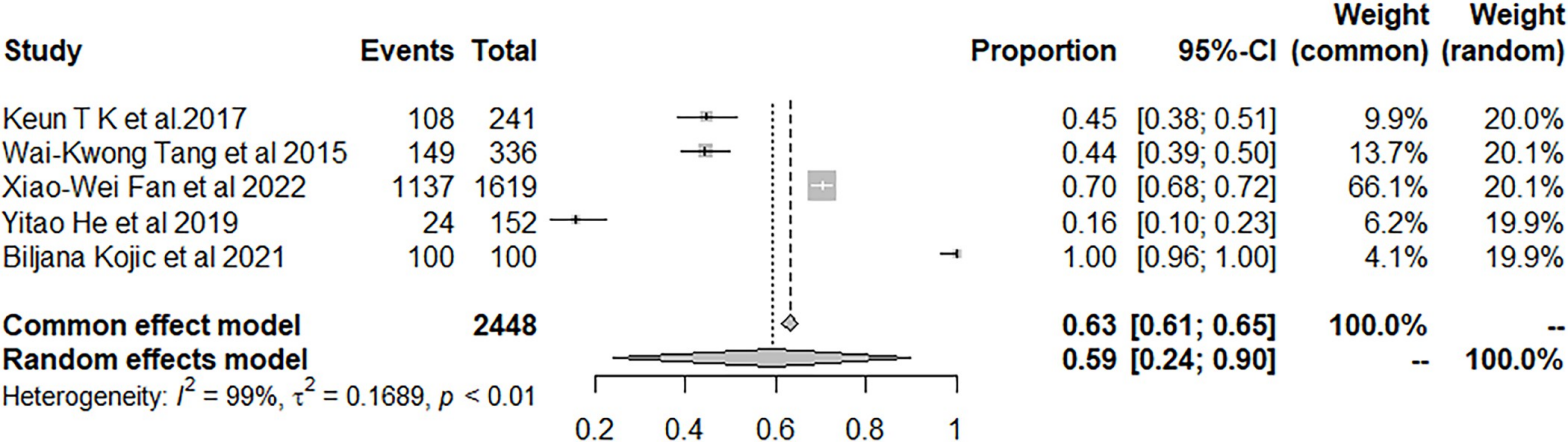
Forest plot of the meta-analysis of prevalence of insomnia among acute stroke stroke patients.

**Fig 7:**
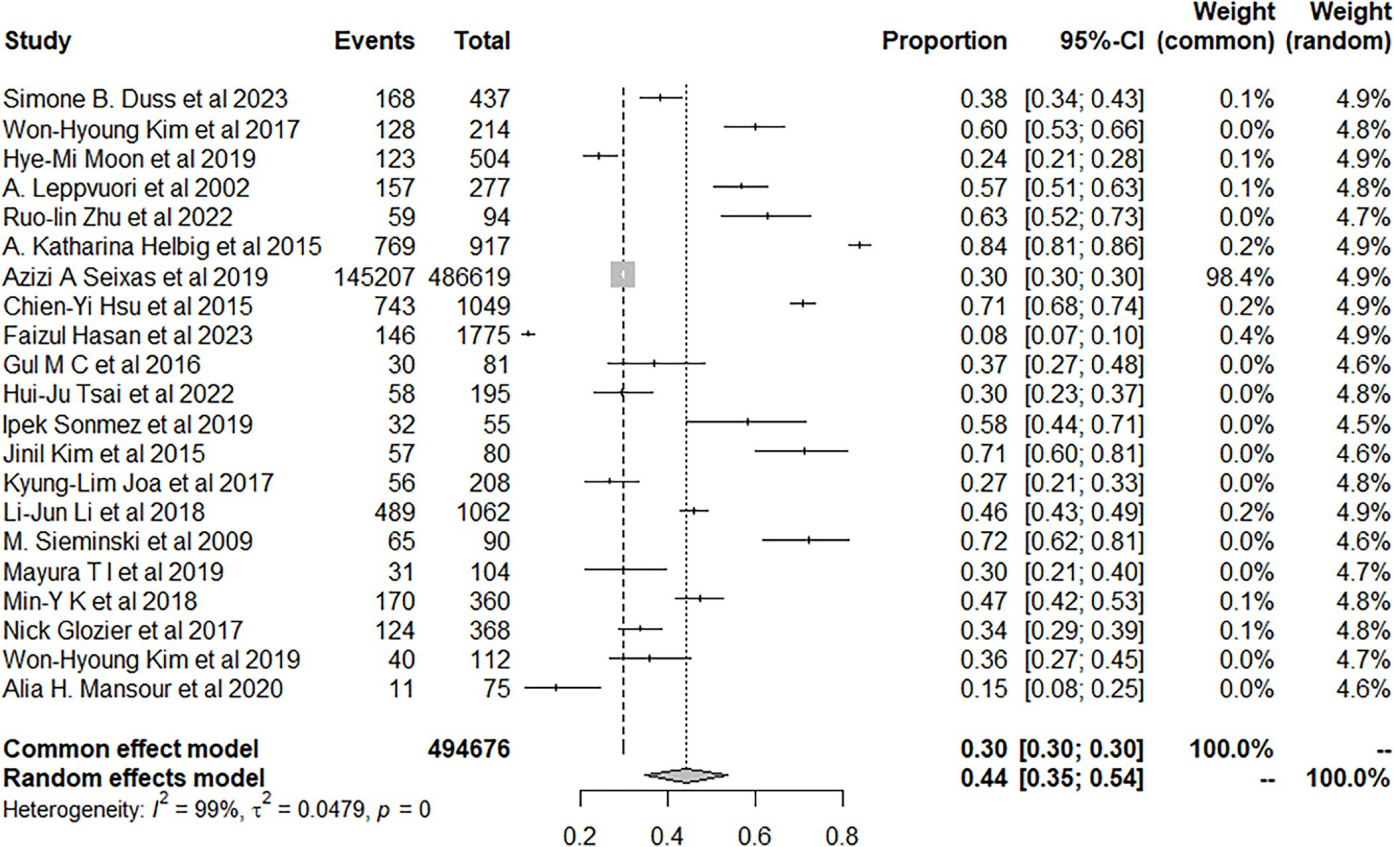
Forest plot of the meta-analysis of prevalence of insomnia among non-acute stroke patients.

Five studies explored the odds of insomnia in patients with acute stroke, and the prevalence was 59.16% (95% CI: 24.18–89.55) (Fig 6). Meanwhile, the odds of insomnia in patients with nonacute stroke was 44.07% in twenty-one studies (95% CI: 34.74–53.61) (Fig 7).

**Fig 8:**
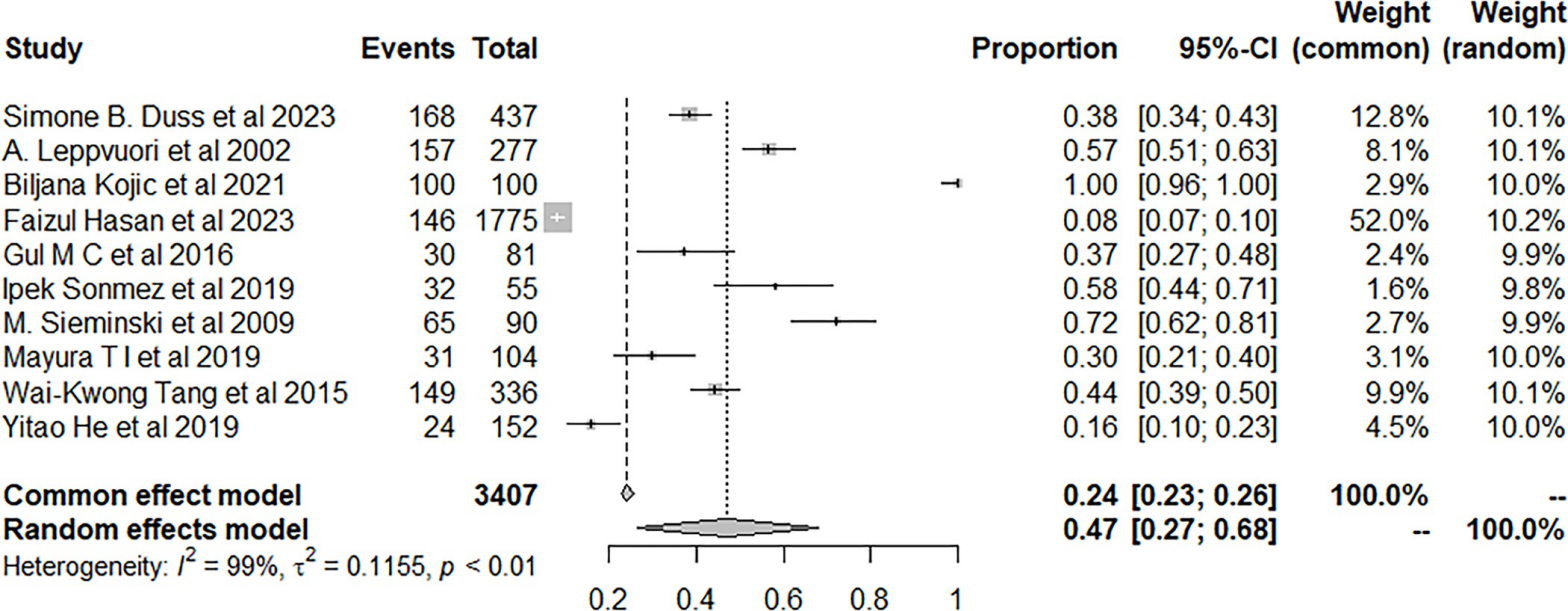
Forest plot of the meta-analysis of prevalence of insomnia in patients of mean age ≥65 years with stroke patients.

**Fig 9:**
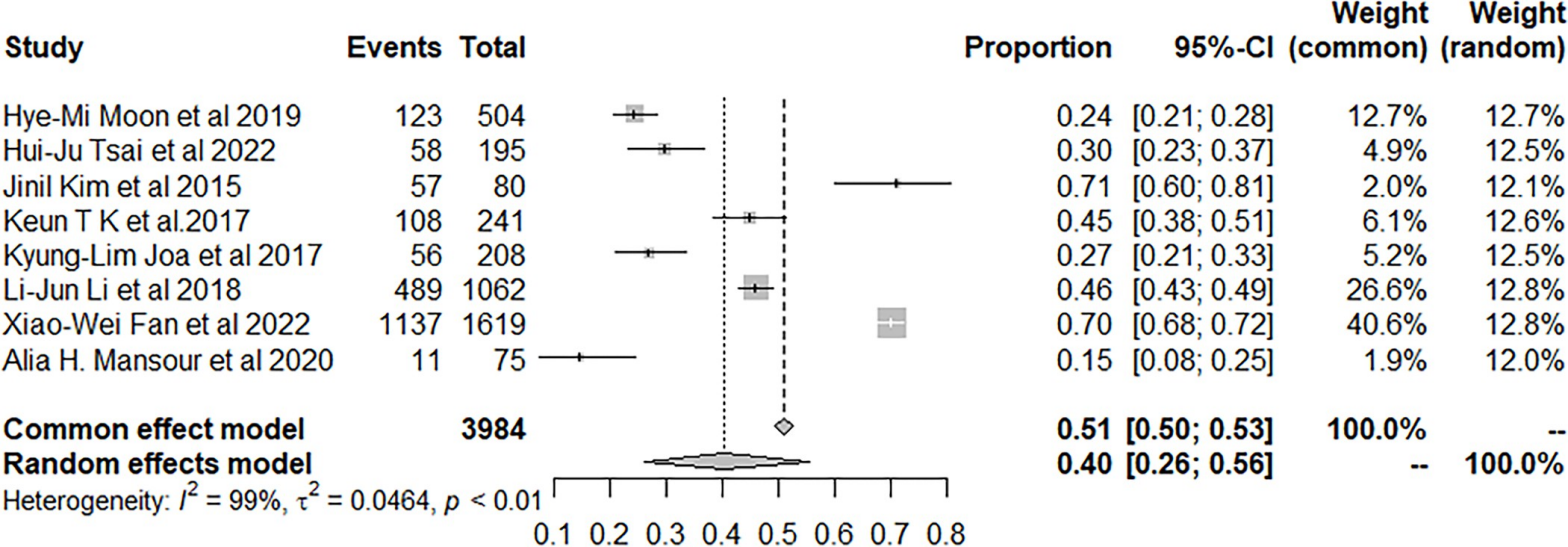
Forest plot of the meta-analysis of prevalence of insomnia in patients of mean age <65 years with stroke patients.

In the subgroup analysis, we found that the incidence of insomnia was significantly higher in the patients with stroke at a mean age of ≥65 than patients with stroke at a mean age of <65 years, which was [47.18% (95% CI: 26.7–68.16) vs 40.50% (95% CI: 26.21–55.66), *P*<0.05] (Figs 8 and 9).

**Fig 10:**
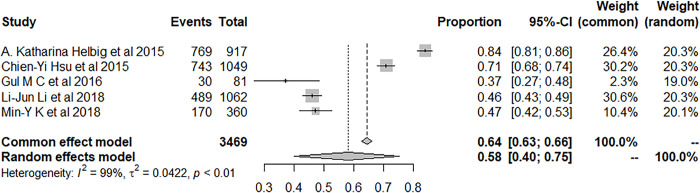
Forest plot of the meta-analysis of prevalence of insomnia among patients with stroke with follow-up for ≥ 3years.

**Fig 11:**
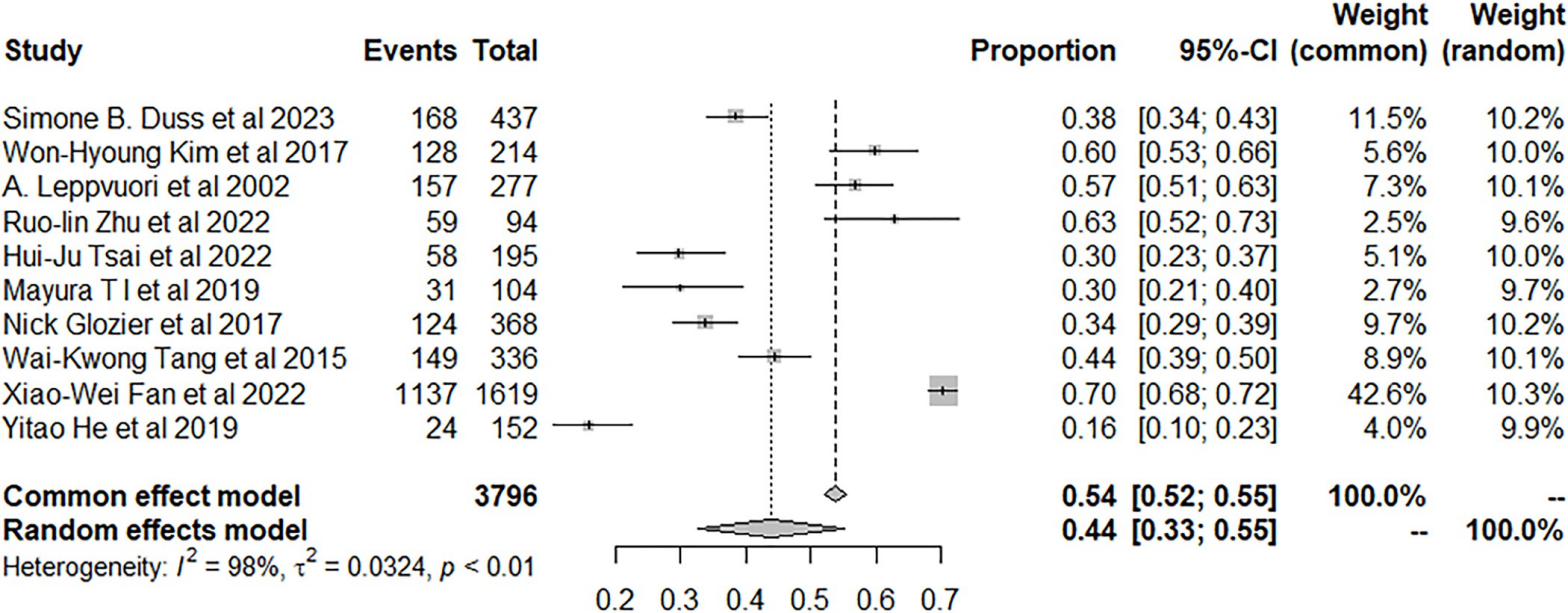
Forest plot of the meta-analysis of prevalence of insomnia among patients with stroke with follow-up for < 3years.

Moreover, concerning the follow-up duration of the participants, we found that the prevalence of insomnia was significantly higher in the follow-up duration was ≥3 years than those with a follow-up period <3 years (58.06% vs 43.83%, *P* < 0.001) (Figs 10 and 11).

**Fig 12:**
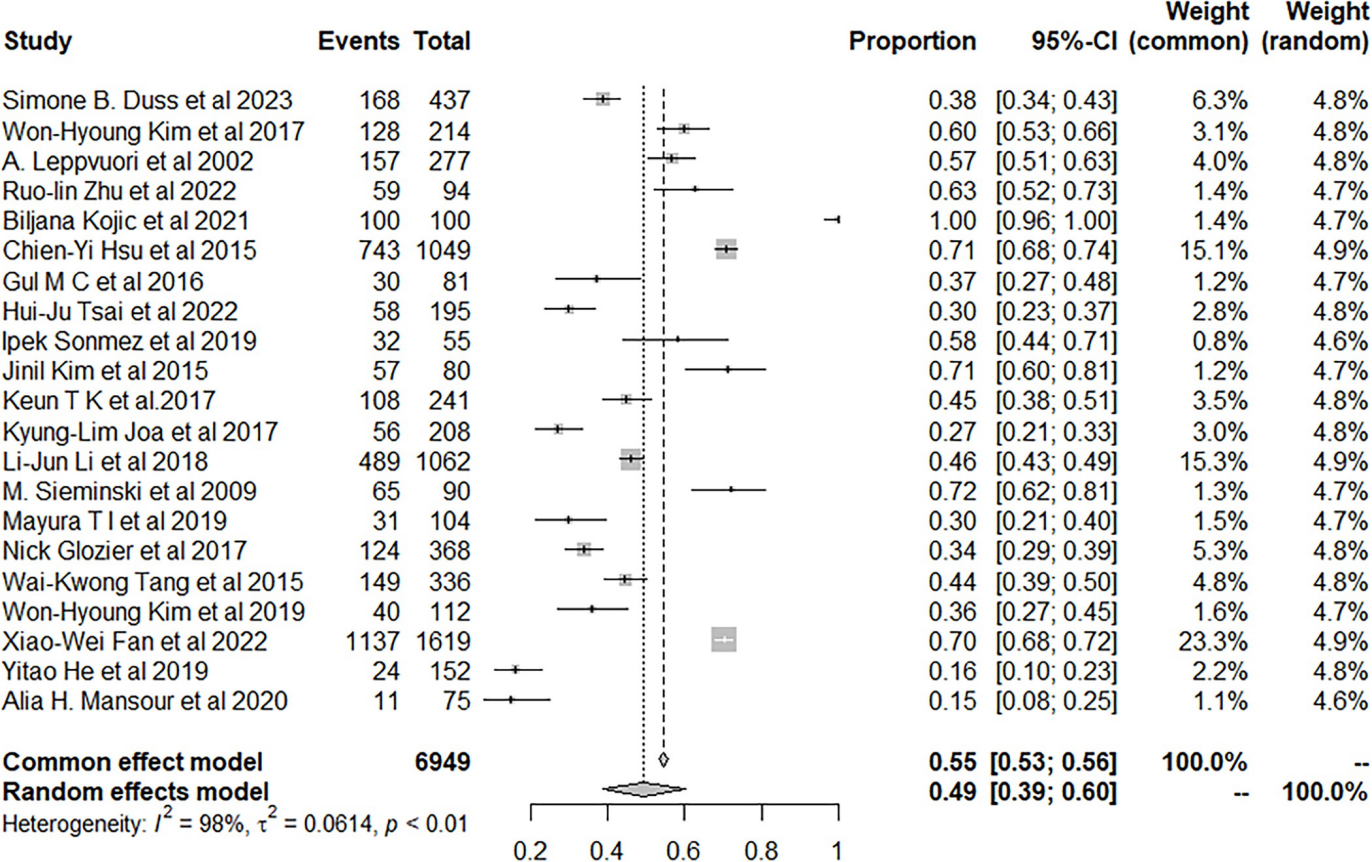
Forest plot of the subgroup analysis with assessment tool.

**Fig 13:**
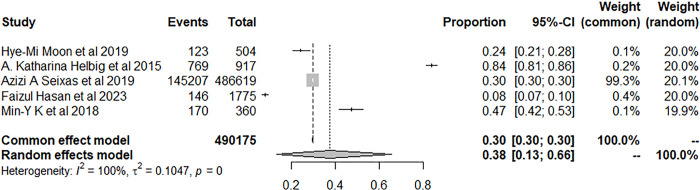
Forest plot of the subgroup analysis without assessment tool.

In the end, the subgroup analyse was performed based on the use of insomnia assessment diagnostic tools (clinical assessment diagnostic tools vs self-report). Twenty-one studies used insomnia assessment diagnostic tools, and the insomnia rate in stroke patients was 49.31% (95% CI: 38.59–60.06) (Fig 12). Five studies used self-report, and the results indicated that the insomnia rate in stroke patients was 37.58% (95% CI: 13.44–65.63) (Fig 13).

## 4 Discussion

### 4.1 Key findings

This study was an updated review about the prevalence of insomnia among patients with stroke. Further, 26 studies from 11 countries were included, of which 15 studies were conducted in Asia (57.69%) and the remaining studies were conducted outside Asia. Of the 26 included studies, 21 used diagnostic tools and 5 used nondiagnostic tools for assessing insomnia.

Overall, our meta-analysis indicated that the rate of insomnia after stroke was 48.37%. It was estimated that incidence of IS or TIA (47.21%) was higher than that of unclassified stroke (41.51%); the rate of acute-phase stroke was higher (59.16%) than that of nonacute-phase stroke (36.31%); the proportion of patients with a mean age ≥65 years was higher (47.18%) than the proportion of those with a mean age <65 years (44.43%); the duration of follow-up ≥3 years (58.06%) was higher than the duration of follow-up <3 years (43.83%); and the rate of using a diagnostic tool for insomnia assessment was higher (51.16%) than the rate of using a nondiagnostic tool (37.58%). This suggested that post-stroke insomnia was a substantial global public health problem in patients with stroke who needed urgent attention for prevention and treatment.

### 4.2 Comparisons of the study findings with the available evidence

Our study found that the rate of insomnia after stroke (48.37%) was 1.27 times higher compared with the prevalence in the meta-analysis by Baylan et al. in 2019 (38.2%) [44]. It indicated that the prevalence of post-stroke insomnia continued to increase yearly, and insomnia had a significant negative impact on patients. The data in this study indicated that sleep-related apnea was significantly associated with stroke, and obstructive apnea syndrome might increase the risk of stroke twice [1]. A 4-year follow-up study in Taiwan, China revealed that compared with patients without insomnia, the incidence of stroke was significantly higher in insomnia patients [45]. A similar meta-analysis showed that sleep duration was also associated with the risk of stroke, with a 5%–7% increase in stroke risk for every 1-h decrease in short sleep duration (RR = 1.05–1.07, 95% CI: 1.01–1.12) [46,47].

Insomnia after stroke is associated with the acute or chronic phase of stroke. In this study, we found that the rate of insomnia was higher in the acute phase of stroke (59.16%) than in the nonacute phase of stroke (36.31%). Luisa et al. found that polysomnography in acute IS patients showed poorer sleep quality was associated with sleep efficiency, sleep-onset awakening time in stroke patients [48]. Several factors usually caused insomnia in patients with stroke. Insomnia in patients with acute stroke was found to be associated with an increased risk of post-stroke psychiatric disorders [49].

Moreover, the age of patients with stroke and the duration of follow-up are also important factors influencing the rate of insomnia in patients with stroke. In the general population, insomnia may increase with age [50]. Studies showed a significantly higher prevalence of insomnia in elderly people [51]. Nick Glozier et al. found that the prevalence of insomnia was 16% after 6 months of stroke and 23% after 12 months of stroke [38]. The aforementioned study suggested that older patients with stroke might have an increased likelihood of experiencing insomnia during the follow-up period, and this likelihood seems to grow over time.

Insomnia assessment and diagnostic tool is also one of the factors affecting the rate of insomnia. This study found that the prevalence of insomnia using the Insomnia Assessment Diagnostic Tool was 51.16%, which was higher than the prevalence of self-reported insomnia (37.58%). In contrast, in study using the insomnia assessment and diagnostic tool, the prevalence of insomnia was different in acute phase and subacute phase stroke (32.5% vs 34.8%), whereas the overall prevalence of self-assessed insomnia also was different in acute phase and subacute phase stroke (47.1% vs 50.4%) [52]. Further large-sample studies are needed to validate these findings.

This study had some limitations. First, the study quality was not an exclusion criterion, which might have contributed to the differences in the prevalence of insomnia after stroke. Studies used different tools for assessing and diagnosing insomnia, which might also have led to biased conclusions. Second, we did not study the treatment of patients with stroke and its effect on the development of insomnia.

## 5 Conclusions

Stroke may be a predisposing factor for insomnia. Insomnia is more likely to occur in acute-phase stroke, and the prevalence of insomnia increases with patient age and follow-up. Further, the rate of insomnia is higher in patients with stroke who use the Insomnia Assessment Diagnostic Tool.

## Supporting information

S1 Checklist PRISMA 2020 checklist. (DOCX)

S1 Data (XLSX)

S1 File (PDF)

## References

[1] Geoffrey A Donnan, Marc Fisher, Macleod Malcolm, Stephen M Davis. Stroke[J].Lancet.2008,371(9624):1612–23. doi: 10.1016/S0140-6736(08)60694-7 18468545

[2] WHO. The Top 10 Causes of Death. Available online: https://www.who.int/news-room/fact-sheets/detail/the-top-10-causesof-death (accessed on 30 January 2022).

[3] Baylan S.; Griffiths S.; Grant N.; Broomfield N.M.; Evans J.J.; Gardani M. Incidence and prevalence of post-stroke insomnia:A systematic review and meta-analysis[J]. Sleep Med. Rev. 2020, 49, 101222. doi: 10.1016/j.smrv.2019.101222 31739180

[4] Baylan Satu, Griffiths Susan, Grant Nicola, Niall M Broomfield, Jonathan J Evans, Maria Gardani. Incidence and prevalence of post-stroke insomnia: A systematic review and meta-analysis[J]. Sleep Med Rev. 2020;49:101222. doi: 10.1016/j.smrv.2019.101222 31739180

[5] American Academy of Sleep Medicine. International classification of Sleep disorders[M]. 3rd ed. Darien, IL:American Academy of Sleep Medicine, 2014.

[6] Pallesen Ståle, Sivertsen Børge, Inger Hilde Nordhus, Bjørn Bjorvatn. A 10-year trend of insomnia prevalence in the adult Norwegian population[J]. Sleep Med. 2014;15(2):173–9. doi: 10.1016/j.sleep.2013.10.009 24382513

[7] Leppävuori T A. Pohjasvaara R. Vataja M. Kaste T. Erkinjuntti. Insomnia in Ischemic Stroke Patients[J]. Cerebrovasc Dis 2002;14:90–97. doi: 10.1159/000064737 12187012

[8] Gottlieb Elie, Landau Elizabeth, Baxter Helen, Werden Emilio, Mark E Howard, Amy Brodtmann. The bidirectional impact of sleep and circadian rhythm dysfunction in human ischaemic stroke: A systematic review[J]. Sleep Med Rev. 2019; 45:54–69. doi: 10.1016/j.smrv.2019.03.003 30954762

[9] Group P–P, Moher D, Shamseer L, Clarke M, Ghersi D, Liberati A, et al. Preferred reporting items for systematic review and meta-analysis protocols (PRISMA-P) 2015 statement. Syst Rev. 2015;4(1):1. doi: 10.1186/2046-4053-4-1 25554246 PMC4320440

[10] Lin Aitao, Tan Yongyi, Chen Jinxia, Liu Xiaoyu, Wu Jinyu. Development of ankylosing spondylitis in patients with ulcerative colitis: A systematic meta-analysis[J]. PLoS One. 2023;18(8):e0289021. doi: 10.1371/journal.pone.0289021 37527250 PMC10393153

[11] Chinese Neurological Association, Chinese Neurosurgical Association, Diagnostic points of various cerebrovascular diseases (1995)[J]. Journal of Clinical and Experimental Medicine, 2013, 7(12):559.

[12] American Academy of Sleep Medicine. International classification of Sleep disorders[M]. 3rd ed. Darien, IL: American Academy of Sleep Medicine, 2014.

[13] Munn Zachary, Moola Sandeep, Riitano Dagmara, Lisy Karolina. The development of a critical appraisal tool for use in systematic reviews addressing questions of prevalence[J]. Int J Health Policy Manag. 2014; 3(3):123–8. doi: 10.15171/ijhpm.2014.71 25197676 PMC4154549

[14] Munn Zachary, Moola Sandeep, Lisy Karolina, Riitano Dagmara, Tufanaru Catalin. Methodological guidance for systematic reviews of observational epidemiological studies reporting prevalence and cumulative incidence data[J]. Int J Evid Based Healthc. 2015;13(3):147–53. doi: 10.1097/XEB.0000000000000054 26317388

[15] Wells G, Shea B, O’connell D et al (2000) The Newcastle-Ottawa Scale (NOS) for assessing the quality of nonrandomised studies in metaanalyses. Eur JEpidemiol 25(9):603–605.

[16] Borenstein M, Hedges L, Higgins J, Rothstein H. Comprehensive metaanalysis version 2. Englewood: Biostat; 2005. p. 104.

[17] Julian P T Higgins, Simon G Thompson, Jonathan J Deeks, Douglas G Altman. Measuring inconsistency in meta-analyses. BMJ. 2003;327(7414):557–60. doi: 10.1136/bmj.327.7414.557 12958120 PMC192859

[18] Borenstein Michael, Larry V Hedges, Julian P T Higgins, Hannah R Rothstein. A basic introduction to fxed-efect and random-efects models for meta-analysis. Res synth methods. 2010;1(2):97–111. doi: 10.1002/jrsm.12 26061376

[19] Simone B Duss, Stefan A Bauer-Gambelli, Corrado Bernasconi, et al. Frequency and evolution of sleep-wake disturbances after ischemic stroke: A 2-year prospective study of 437 patients[J]. Sleep Med. 2023;101:244–251. doi: 10.1016/j.sleep.2022.10.007 36446142

[20] Kim Won-Hyoung, Jung Han-Young, Choi Ha-Yoon, Park Chan-Hyuk, Kim Eun-Suk, Lee Sook-Joung, et al. The associations between insomnia and health-related quality of life in rehabilitation units at 1month after stroke[J]. J Psychosom Res. 2017;96:10–14. doi: 10.1016/j.jpsychores.2017.02.008 28545786

[21] Moon Hye-Mi, Kim Yoonjung. Mental health according to sleep duration in stroke survivors: A population-based nationwide cross-sectional study[J]. Geriatr Gerontol Int. 2020;20(3):223–228. doi: 10.1111/ggi.13846 31837251

[22] Zhu Ruo-Lin, Ouyang Chao, Ma Ruo-Lin, Wang Kai. Obstructive sleep apnea is associated with cognitive impairment in minor ischemic stroke[J]. Sleep Breath. 2022;26(4):1907–1914. doi: 10.1007/s11325-022-02575-5 35305233 PMC9663369

[23] A Katharina Helbig Doris Stöckl, Heier Margit, Ladwig Karl-Heinz, Meisinger Christa. Symptoms of Insomnia and Sleep Duration and Their Association with Incident Strokes: Findings from the Population-Based MONICA/KORA Augsburg Cohort Study[J]. PLoS One. 2015 31;10(7):e0134480. doi: 10.1371/journal.pone.0134480 26230576 PMC4521822

[24] Azizi A Seixas, Debbie P Chung, Shannique L Richards, Madhavaram Shreya, Preeti Raghavan, Juan Gago, et al. The impact of short and long sleep duration on instrumental activities of daily living among stroke survivors[J]. Neuropsychiatr Dis Treat. 2019;15:177–182. doi: 10.2147/NDT.S177527 30655670 PMC6324604

[25] Biljana Kojic, Zikrija Dostovic, Omer C Ibrahimagic, Dzevdet Smajlovic, Renata Hodzic, Amra Iljazovic, et al. Risk Factors in Acute Stroke Patients With and Without Sleep Apnea[J]. Med Arch. 2021;75(6):444–450. doi: 10.5455/medarh.2021.75.444–450 35169372 PMC8802685

[26] Hsu Chien-Yi, Chen Yung-Tai, Chen Mu-Hong, Huang Chin-Chou, et al. The Association Between Insomnia and Increased Future Cardiovascular Events: A Nationwide Population-Based Study[J]. Psychosom Med. 2015;77(7):743–51. doi: 10.1097/PSY.0000000000000199 26355726

[27] Faizul Hasan, Muhtar M S, Dean Wu, Hsin-Chien Lee Yen-Chun Fan, Ting-Jhen Chen, et al. Post-Stroke Insomnia Increased the Risk of Cognitive Impairments: A Hospital-Based Retrospective Cohort Study[J]. Behav Sleep Med. 2023;1–9. doi: 10.1080/15402002.2023.2165491 36606311

[28] Gul Mete Civelek, Ayce Atalay, Turhan Nur. Medical complications experienced by first-time ischemic stroke patients during inpatient, tertiary level stroke rehabilitation[J]. J Phys Ther Sci. 2016;28(2):382–91. doi: 10.1589/jpts.28.382 27065523 PMC4792978

[29] Tsai Hui-Ju, Wong Yi-Sin, Ong Cheung-Ter. Hui-Ju Tsai 1, Yi-Sin Wong 2, Cheung-Ter Ong[J]. PLoS One. 2022;17(11):e0277309. doi: 10.1371/journal.pone.0277309 36346797 PMC9642877

[30] Sonmez Ipek, Karasel Seide. Poor Sleep Quality I Related to Impaired Functional Status Following Stroke[J]. J Stroke Cerebrovasc Dis. 2019;28(11):104349. doi: 10.1016/j.jstrokecerebrovasdis.2019.104349 31492629

[31] Kim Jinil, Kim Yuntae, Kwang Ik Yang Doh-Eui Kim, Soo A Kim. The Relationship Between Sleep Disturbance and Functional Status in Mild Stroke Patients[J]. Ann Rehabil Med. 2015;39(4):545–52. doi: 10.5535/arm.2015.39.4.545 26361590 PMC4564701

[32] Kim Keun Tae, Moon Hye-Jin, Yang Jun-Gyu, Sohn Sung-Ii, Hong Jeong-Ho, Cho Yong Won. The prevalence and clinical significance of sleep disorders in acute ischemic stroke patients-a questionnaire study[J]. Sleep Breath. 2017;21(3):759–765. doi: 10.1007/s11325-016-1454-5 28064431

[33] Joa Kyung-Lim, Kim Won-Hyoung, Choi Ha-Yoon, Park Chan-Hyuk, Kim Eun-Suk, Lee Sook-Joung, et al. The Effect of Sleep Disturbances on the Functional Recovery of Rehabilitation Inpatients Following Mild and Moderate Stroke[J]. Am J Phys Med Rehabil. 2017;96(10):734–740. doi: 10.1097/PHM.0000000000000744 28368898

[34] Li Li-Jun, Yang Yang, Guan Bo-Yuan, Chen Qi, Wang An-Xin, Wang Yong-Jun, et al. Insomnia is associated with increased mortality in patients with first-ever stroke: a 6-year follow-up in a Chinese cohort study[J]. Stroke Vasc Neurol. 2018;3(4):197–202. doi: 10.1136/svn-2017-000136 30637124 PMC6312128

[35] Sieminski M., Chwojnicki K., Ossowska A., Wierucki L., Zdrojewski T., Wyrzykowski B., et al. Impact of insomnia on the quality of life of post-stroke patients[J]. 19th World Congress of Neurology, Free Paper Abstracts / Journal of the Neurological Sciences 285 S1 (2009) S57–S154.

[36] Mayura T Iddagoda, Charles A Inderjeeth, Kien Chan, Warren D Raymond. Post-stroke sleep disturbances and rehabilitation outcomes: a prospective cohort study[J]. Intern Med J. 2020;50(2):208–213. doi: 10.1111/imj.14372 31111660

[37] Kim Min-Young, Lee Seunghoon, You Ho Myong Yoon Jae Lee, Kim Me-Riong, Shin Joon-Shik, et al. Association between sleep duration and stroke prevalence in Korean adults: a cross-sectional study[J]. BMJ Open. 2018;8(6):e021491. doi: 10.1136/bmjopen-2018-021491 29903797 PMC6009631

[38] Glozier Nick, Tom J Moullaali Børge Sivertsen, Kim Dukyeon, Mead Gillian, Jan Stephen, et al. The Course and Impact of Poststroke Insomnia in Stroke Survivors Aged 18 to 65 Years: Results from the Psychosocial Outcomes In StrokE (POISE) Study[J]. Cerebrovasc Dis Extra. 2017;7(1):9–20. doi: 10.1159/000455751 28161702 PMC5346918

[39] Tang Wai-Kwong, Lau Chieh Grace, Mok Vincent, Ungvari Gabor S, Wong Ka-Sing. Insomnia and health-related quality of life in stroke[J]. Top Stroke Rehabil. 2015;22(3):201–7. doi: 10.1179/1074935714Z.0000000026 25908494

[40] Kim Won-Hyoung, Yoo Young-Hwan, Lim Ju-Young, Kang Sang-Gu, Jung Han-Young, Bae Jae Nam, et al. Objective and subjective sleep problems and quality of life of rehabilitation in patients with mild to moderate stroke[J]. Top Stroke Rehabil. 2020;27(3):199–207. doi: 10.1080/10749357.2019.1673591 31618116

[41] Fan Xiao-Wei, Yang Yang, Wang Shuo, Zhang Yi-Jun, Wang An-Xin, Liao Xiao-Ling, et al. Impact of Persistent Poor Sleep Quality on Post-Stroke Anxiety and Depression: A National Prospective Clinical Registry Study[J]. Nat Sci Sleep. 2022;14:1125–1135. doi: 10.2147/NSS.S357536 35721879 PMC9205438

[42] He Yitao, Gu Mei, Zhang Hui, Deng Jian, Wu Xiaoyun, Guo Yi. Effect of insomnia after acute ischemic stroke on cerebrovascular reactivity: a prospective clinical study in China[J]. Sleep Med. 2019;63:82–87. doi: 10.1016/j.sleep.2019.07.005 31606653

[43] Mansour Alia H., Ayad Maged, Naglaa El-Khayat, Ahmed El Sadekand Alloush Taha K. Post-stroke sleep disorders in Egyptian patients by using simply administered questionnaires: a study from Ain Shams University[J]. The Egyptian Journal of Neurology, (2020) 56:13. doi: 10.1186/s41983-020-0148-x

[44] Baylan Satu, Griffiths Susan, Grant Nicola, Niall M Broomfield, Jonathan J Evans, Maria Gardani. Incidence and prevalence of post-stroke insomnia: A systematic review and meta-analysis[J]. Sleep Med Rev. 2020;49:101222. doi: 10.1016/j.smrv.2019.101222 31739180

[45] Wu Ming-Ping, Lin Huey-Juan, Weng Shih-Feng, Ho Chung-Han, Wang Jhi-Joung, Hsu Ya-Wen. Insomnia subtypes and the subsequent risks of stroke: report from a nationally representative cohort[J]. Stroke. 2014;45(5):1349–54. doi: 10.1161/STROKEAHA.113.003675 24699057

[46] Simone B Duss Anne-Kathrin Brill, Bargiotas Panagiotis, Facchin Laura, Alexiev Filip, Manconi Mauro, et al. Sleep-Wake Disorders in Stroke-Increased Stroke Risk and Deteriorated Recovery? An Evaluation on the Necessity for Prevention and Treatment[J]. Curr Neurol Neurosci Rep. 2018;18(10):72. doi: 10.1007/s11910-018-0879-6 30194550

[47] Li Wenzhen, Wang Dongming, Cao Shiyi, Yin Xiaoxv, Gong Yanhong, Gan Yong, et al. Sleep duration and risk of stroke events and stroke mortality: A systematic review and meta-analysis of prospective cohort studies[J]. Int J Cardiol. 2016;223:870–876. doi: 10.1016/j.ijcard.2016.08.302 27584562

[48] Luisa de Vivo, Michele Bellesi, Marshall William, Eric A Bushong, Mark H Ellisman, Giulio Tononi, et al. Ultrastructural evidence for synaptic scaling across the wake/sleep cycle[J]. Science. 2017;355(6324):507–510. doi: 10.1126/science.aah5982 28154076 PMC5313037

[49] Nakamizo Tomoki, Kanda Toshie, Kudo Yosuke, Sugawara Eriko, Hashimoto Erina, Okazaki Ayana, et al. Effects of uncomfortable care and histamine H2-antagonists on delirium in acute stroke: A propensity score analysis[J]. J Neurol Sci. 2021;420:117251. doi: 10.1016/j.jns.2020.117251 33276246

[50] Leino Akseli, Susanna Westeren-Punnonen Juha Töyräs, et al. Acute stroke and TIA patients have specific polygraphic features of obstructive sleep apnea[J]. Sleep Breath. 2020;24(4):1495–1505. doi: 10.1007/s11325-019-02010-2 31938989 PMC7679322

[51] Benbir Gulcin, Ahmet Ugur Demir, Murat Aksu, Ardic Sadik, Firat Hikmet, Itil Oya, et al. Prevalence of insomnia and its clinical correlates in a general population in Turkey[J]. Psychiatry Clin Neurosci. 2015;69(9):543–52. doi: 10.1111/pcn.12252 25384688

[52] Rollo Eleonora, Brunetti Valerio, Scala Irene, Callea Antonio, Marotta Jessica, Vollono Catello, et al. Impact of delirium on the outcome of stroke: a prospective, observational, cohort study[J]. J Neurol. 2022;269(12):6467–6475. doi: 10.1007/s00415-022-11309-2 35945396 PMC9618551

