# Supplementary material for: Development of insomnia in patients with stroke: A systematic review and meta-analysis
Source: PLoS One. 2024 Apr 10;19(4):e0297941. doi: 10.1371/journal.pone.0297941 (PMC11006172; doi:10.1371/journal.pone.0297941)
Supplement: S1 Checklist — (DOCX) [file pone.0297941.s001.docx]

| **Section and Topic** | **Item #** | **Checklist item** | **Location where item is reported** |
| --- | --- | --- | --- |
| **TITLE** | | |  |
| Title | 1 | Identify the report as a systematic review. | **In a review investigating the development of** **Insomnia in patients with Stroke, the authors identify the report as a systematic review**: “Development of Insomnia in patients with Stroke: A systematic review and meta-analysis”. |
| **ABSTRACT** | | |  |
| Abstract | 2 | See the PRISMA 2020 for Abstracts checklist. | **In a review investigating the development of Insomnia in patients with stroke, the authors summarise the objectives, eligibility criteria, databases consulted, methods for collecting data and synthesising results, along with presenting results and funding source for the review.**  “**Background and aim：**Stroke is a serious threat to human life and health, and post-stroke insomnia is one of the common complications severely impairing patients' quality of life and delaying recovery. Early understanding of the relationship between stroke and post-stroke insomnia can provide clinical evidence for preventing and treating post-stroke insomnia. This study was to investigate the prevalence of insomnia in patients with stroke.  **Methods:** The Web of Science, PubMed, Embase, and Cochrane Library databases were used to obtain the eligible studies until June 2023. The quality assessment was performed to extract valid data for meta-analysis.  The prevalence rates were used a random-efect. I2 statistics were used to assess the heterogeneity of the studies.  **Results:** (1) Twenty-six studies met the inclusion criteria for meta-analysis, with 1,193,659 participants, of which 497,124 were patients with stroke.  (2) The meta-analysis indicated that 150,181 patients with stroke developed insomnia during follow-up [46.98%, 95% confidence interval (CI): 36.91–57.18] and 1806 patients with ischemic stroke (IS) or transient ischemic attack (TIA) developed insomnia (47.21%, 95% CI: 34.26–60.36). Notably, 41.51% of patients with the prevalence of nonclassified stroke developed insomnia (95% CI: 28.86–54.75). The incidence of insomnia was significantly higher in patients with acute strokes than in patients with nonacute strokes (59.16% vs 44.07%, P < 0.0001).  (3) Similarly, the incidence of insomnia was significantly higher in the patients with stroke at a mean age of ≥65 than patients with stroke at a mean age of <65 years (47.18% vs 40.50%, P < 0.05). Fifteen studies reported the follow-up time. The incidence of insomnia was significantly higher in the follow-up for ≥3 years than follow-up for <3 years (58.06% vs 43.83%, P < 0.05). Twenty-one studies used the Insomnia Assessment Diagnostic Tool, and the rate of insomnia in patients with stroke was 49.31% (95% CI: 38.59–60.06). Five studies used self-reporting, that the rate of insomnia in patients with stroke was 37.58% (95% CI: 13.44–65.63).  **Conclusions:** Stroke may be a predisposing factor for insomnia. Insomnia is more likely to occur in acute-phase stroke, and the prevalence of insomnia increases with patient age and follow-up time. Further, the rate of insomnia is higher in patients with stroke who use the Insomnia Assessment Diagnostic Tool.  **Funding:** This work was supported by Administration of Traditional Chinese medicine in guangxi, self-financing scientific research subject[grant numbers GXZYA20220072]; National Natural Science Foundation of China [grant numbers 2022JJA141329]; Hospital scientific research project of the First Affiliated Hospital of Guangxi University of Traditional Chinese Medicine[grant numbers 2021QN008]; Guangxi University of Traditional Chinese Medicine research project[grant numbers 2022QN019]”. |
| **INTRODUCTION** | | |  |
| Rationale | 3 | Describe the rationale for the review in the context of existing knowledge. | **In a review investigating the development of** **Insomnia in patients with stroke, the authors report what information the review seeks to add to current knowledge, and indicate that no systematic review addressing the same question exists:**  “Stroke is the second most morbid and deadly disease globally, which is characterized by high morbidity, disability, mortality, and recurrence. It substantially threatens human life, health, and quality of life[1-2]. Neuropsychiatric disorders frequently affect stroke survivors, with about one third suffering from mental health problems such as insomnia, depression, or anxiety[3]. Studies have found that approximately one third of patients after stroke meet diagnostic criteria for insomnia, and patients may experience difficulty falling asleep, difficulty with sleep persistence, and early awakening[4].  Insomnia is the most common sleep disorder prevalent in people of all ages. In severe cases, it can affect daytime work and life, and even cause emotional disorders[5]. The incidence of insomnia increases with the increase in social pressure[6]. Patients with stroke are significantly more likely to suffer from insomnia than the normal healthy population, and some patients with insomnia may be more prone to stroke risk[7]. Insomnia is likely to be an independent risk factor for stroke, as increasing clinical studies have found a bidirectional relationship between insomnia and stroke. Further, stroke may also be a predisposing factor for insomnia[8]. Therefore, it is essential to understand the relationship between stroke and post-stroke insomnia in an early stage to provide a clinical basis for the early prevention and treatment of post-stroke insomnia. In this study, we conducted a systematic literature review and meta-analysis to assess the incidence of insomnia and associated risk factors in patients with stroke.” |
| Objectives | 4 | Provide an explicit statement of the objective(s) or question(s) the review addresses. | **In a review investigating the development of Insomnia in patients with stroke, the authors report a single objective of the review:**  “In this study, we conducted a literature search and meta-analysis to investigate the prevalence of insomnia among stroke patients during follow-up.” |
| **METHODS** | | |  |
| Eligibility criteria | 5 | Specify the inclusion and exclusion criteria for the review and how studies were grouped for the syntheses. | **In a review investigating the development of Insomnia in patients with stroke, the authors report the types of studies, participants, and outcomes that were eligible for inclusion in the review, and state that there were restrictions on the type of reports that were eligible (i.e. published, English language):**  “Object of study: Patients with a definite diagnosis of stroke who developed insomnia during the follow-up period were included in the study. Stroke patients met the diagnostic criteria of the Essentials of Diagnosis of Various Cerebrovascular Diseases [10]. Insomnia patients were diagnosed through recognized assessment tools such as the Pittsburgh Sleep Quality Index (PSQI), Hamilton Depression Scale (HDS), Epworth Sleepiness Scale (ESS), or self-reported symptoms of insomnia and met the diagnostic criteria of the American Academy of Sleep in 2014. Types of research and language of publication: English language literature (including cross-sectional studies, cohort studies, case-control studies, etc.) on the occurrence of insomnia during stroke follow-up were included. Exclusion criteria: ① Reviews, case reports, animal and cell-based experiments, etc.). ② Repeatedly included literature, literature with incomplete data indicators or where complete information was not available. |
| Information sources | 6 | Specify all databases, registers, websites, organisations, reference lists and other sources searched or consulted to identify studies. Specify the date when each source was last searched or consulted. | **In a review investigating the development of Insomnia in patients with stroke, the authors report the databases and other sources consulted, along with the date each source was searched:**  “Literature related to the occurrence of developmental insomnia in stroke patients was collected through PubMed, The Cochrane Library, Web of Science, and Embase databases until June 2023. PROSPERO registration number: CRD42023452419.” |
| Search strategy | 7 | Present the full search strategies for all databases, registers and websites, including any filters and limits used. | **In a review investigating the development of Insomnia in patients with stroke, the authors report the full search strategy for Pubmed, along with the list of terms used when searching four websites.**  “Stroke”, “Cerebrovascular Accident”, “Insomnia”, “Insomnia Disorder”, etc. were used as subject terms to search related literature. The logical relationship between subject terms is “and”, and the logical relationship between subject terms and free search terms is “or”. We used the following search strategy for the PubMed database (Fig 1. Search strategy of PubMed.).”  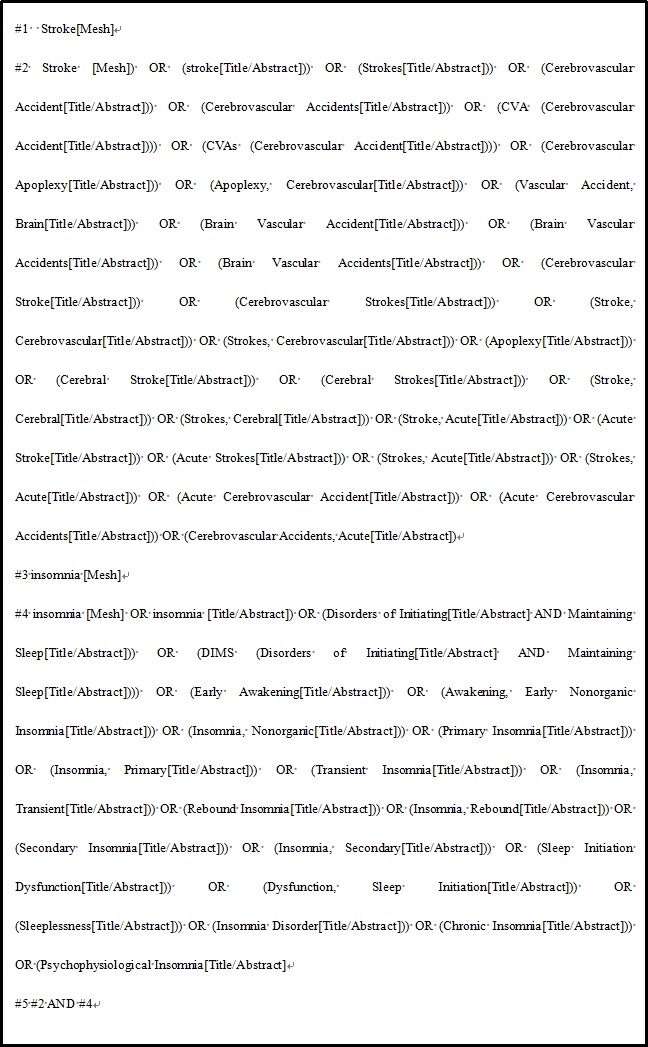 |
| Selection process | 8 | Specify the methods used to decide whether a study met the inclusion criteria of the review, including how many reviewers screened each record and each report retrieved, whether they worked independently, and if applicable, details of automation tools used in the process. | **In a review investigating the development of Insomnia in patients with stroke, the authors report piloting, double screening, and consensus methods for study selection:**  “Two independent researchers (LAT), (TQJ) screened and extracted relevant data from the included studies. The extracted information mainly included the basic information of the literature: first author name, the time of publication, sample size, the country, the follow-up time and the number of positive cases. In case of disagreement between two researchers in the literature screening or data extraction process, the decision was submitted to the third researcher (YJW).” |
| Data collection process | 9 | Specify the methods used to collect data from reports, including how many reviewers collected data from each report, whether they worked independently, any processes for obtaining or confirming data from study investigators, and if applicable, details of automation tools used in the process. | **In a review investigating the development of Insomnia in patients with stroke, the authors report** **using a standardized form, and indicate that independent reviewers extracted data, which was checked by another reviewer:**  “The methodological quality of the included studies was assessed using the Critical Appraisal Tool for Prevalence Studies [12-13]. If 2 researchers disagreed on the assessment of the quality of the literature, the decision was submitted to a third researcher (YJW).” |
| Data items | 10a | List and define all outcomes for which data were sought. Specify whether all results that were compatible with each outcome domain in each study were sought (e.g. for all measures, time points, analyses), and if not, the methods used to decide which results to collect. | **In a review investigating the development of Insomnia in patients with stroke, the authors define the outcomes for which data were sought (e.g.Studies were included that patients with a clear diagnosis of UC who developed AS during follow-up), and specify the decision rules used to decide which results to collect when multiple were available in studies:**  “Patients with a definite diagnosis of stroke who developed insomnia during the follow-up period were included in the study. Stroke patients met the diagnostic criteria of the Essentials of Diagnosis of Various Cerebrovascular Diseases [10]. Insomnia patients were diagnosed through recognized assessment tools such as the Pittsburgh Sleep Quality Index (PSQI), Hamilton Depression Scale (HDS), Epworth Sleepiness Scale (ESS), or self-reported symptoms of insomnia and met the diagnostic criteria of the American Academy of Sleep in 2014 [11].” |
|  | 10b | List and define all other variables for which data were sought (e.g. participant and intervention characteristics, funding sources). Describe any assumptions made about any missing or unclear information. | **In a review investigating the development of Insomnia in patients with stroke, the authors list and define all variables for which data were sought, including characteristics of the study design, participants, and followup:**  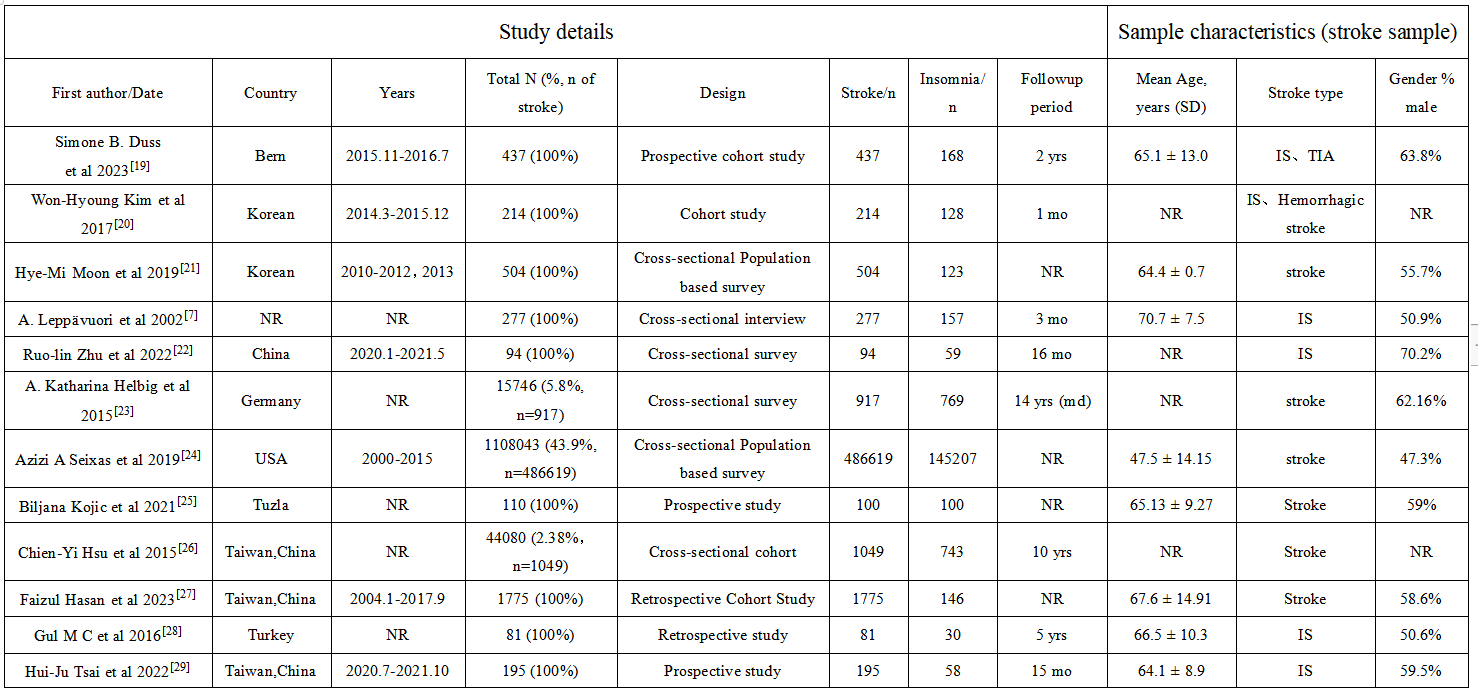  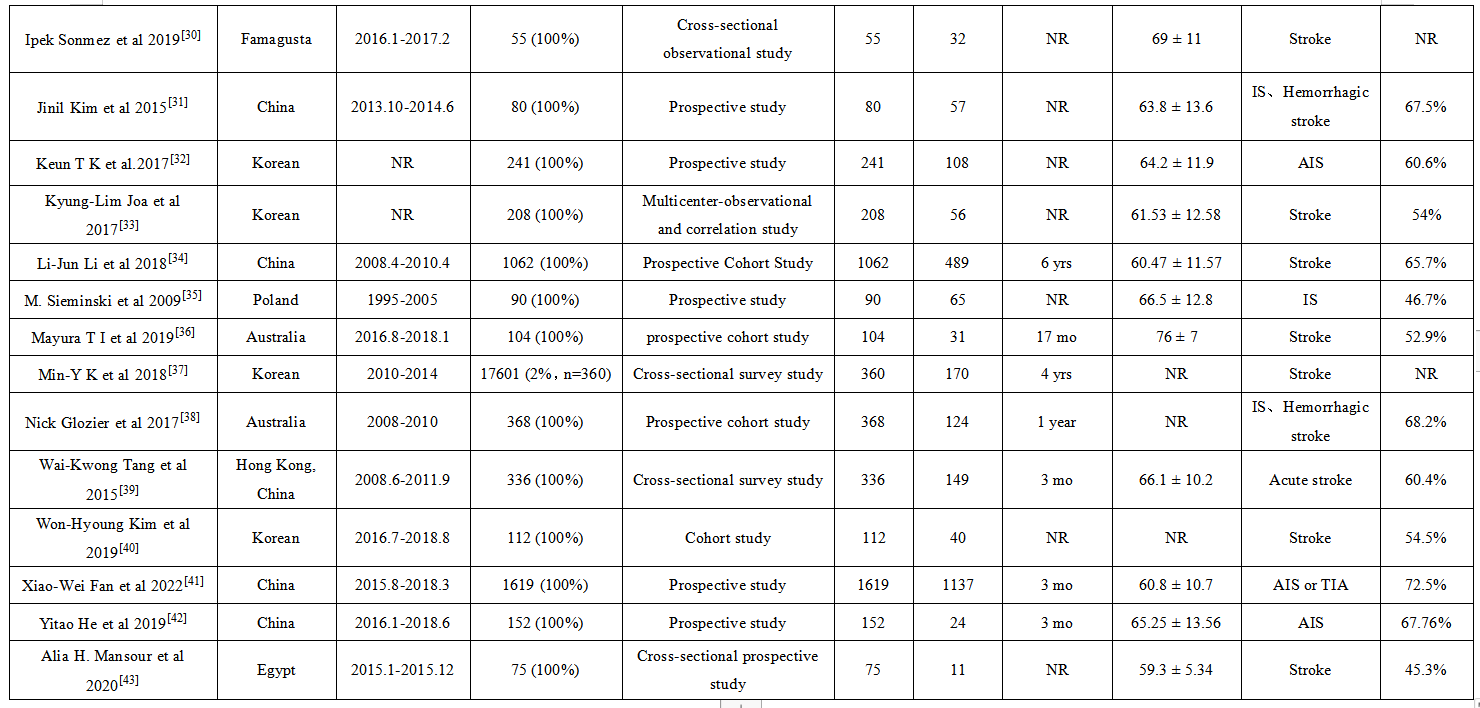 |
| Study risk of bias assessment | 11 | Specify the methods used to assess risk of bias in the included studies, including details of the tool(s) used, how many reviewers assessed each study and whether they worked independently, and if applicable, details of automation tools used in the process. | **In a review investigating the development of Insomnia in patients with stroke, the authors specify the risk of bias tool used, the domains of bias addressed by the tool, how many reviewers assessment each study and how an overall judgement was reached:**  “The methodological quality of the included studies was assessed using the Critical Appraisal Tool for Prevalence Studies [12-13]. If 2 researchers disagreed on the assessment of the quality of the literature, the decision was submitted to a third researcher (YJW).” |
| Effect measures | 12 | Specify for each outcome the effect measure(s) (e.g. risk ratio, mean difference) used in the synthesis or presentation of results. | **In a review investigating the development of Insomnia in patients with stroke, the authors report using the risk ratio in the synthesis or presentation of results for dichotomous outcomes:**  “In the study, we used systematic Meta-Analysis software version 3 to calculate the statistical analyse [15]. The fixed effects model was used in P≥0.10 and I2≤50%, and random-effects model was used in P<0.10 and/or I2>50%, which was necessary to find the source of heterogeneity and perform subgroup analysis or sensitivity analysis [16-18].” |
| Synthesis methods | 13a | Describe the processes used to decide which studies were eligible for each synthesis (e.g. tabulating the study intervention characteristics and comparing against the planned groups for each synthesis (item #5)). | **In a review investigating the development of Insomnia in patients with stroke, the authors report categorising the interventions delivered in the included studies according to guiding:**  “Patients with a definite diagnosis of stroke who developed insomnia during the follow-up period were included in the study. Stroke patients met the diagnostic criteria of the Essentials of Diagnosis of Various Cerebrovascular Diseases [10]. Insomnia patients were diagnosed through recognized assessment tools such as the Pittsburgh Sleep Quality Index (PSQI), Hamilton Depression Scale (HDS), Epworth Sleepiness Scale (ESS), or self-reported symptoms of insomnia and met the diagnostic criteria of the American Academy of Sleep in 2014 [11].” |
|  | 13b | Describe any methods required to prepare the data for presentation or synthesis, such as handling of missing summary statistics, or data conversions. | **In a review investigating the development of Insomnia in patients with stroke, the authors report estimating development of insomnia in stroke patients:**  “In the study, we used systematic Meta-Analysis software version 3 to calculate the statistical analyse [15]. The fixed effects model was used in P≥0.10 and I2≤50%, and random-effects model was used in P<0.10 and/or I2>50%, which was necessary to find the source of heterogeneity and perform subgroup analysis or sensitivity analysis [16-18].” |
|  | 13c | Describe any methods used to tabulate or visually display results of individual studies and syntheses. | **In a review investigating the development of Insomnia in patients with stroke, the authors describe using ‘Summary of findings’ tables to present the synthesis results:**  “(1) Twenty-six studies met the inclusion criteria for meta-analysis, with 1,193,659 participants, of which 497,124 were patients with stroke.  (2) The meta-analysis indicated that 150,181 patients with stroke developed insomnia during follow-up [46.98%, 95% confidence interval (CI): 36.91–57.18] and 1806 patients with ischemic stroke (IS) or transient ischemic attack (TIA) developed insomnia (47.21%, 95% CI: 34.26–60.36). Notably, 41.51% of patients with the prevalence of nonclassified stroke developed insomnia (95% CI: 28.86–54.75). The incidence of insomnia was significantly higher in patients with acute strokes than in patients with nonacute strokes (59.16% vs 44.07%, P < 0.0001).  (3) Similarly, the incidence of insomnia was significantly higher in the patients with stroke at a mean age of ≥65 than patients with stroke at a mean age of <65 years (47.18% vs 40.50%, P < 0.05). Fifteen studies reported the follow-up time. The incidence of insomnia was significantly higher in the follow-up for ≥3 years than follow-up for <3 years (58.06% vs 43.83%, P < 0.05). Twenty-one studies used the Insomnia Assessment Diagnostic Tool, and the rate of insomnia in patients with stroke was 49.31% (95% CI: 38.59–60.06). Five studies used self-reporting, that the rate of insomnia in patients with stroke was 37.58% (95% CI: 13.44–65.63).” |
|  | 13d | Describe any methods used to synthesize results and provide a rationale for the choice(s). If meta-analysis was performed, describe the model(s), method(s) to identify the presence and extent of statistical heterogeneity, and software package(s) used. | **In a review investigating the development of Insomnia in patients with stroke, the authors report their chosen meta-analysis model, along with a rationale, the between-study variance estimator used, methods used to quantify statistical heterogeneity, and the software packages used:**  “In the study, we used systematic Meta-Analysis software version 3 to calculate the statistical analyse [15]. The fixed effects model was used in P≥0.10 and I2≤50%, and random-effects model was used in P<0.10 and/or I2>50%, which was necessary to find the source of heterogeneity and perform subgroup analysis or sensitivity analysis [16-18].” |
|  | 13e | Describe any methods used to explore possible causes of heterogeneity among study results (e.g. subgroup analysis, meta-regression). | **In a review investigating the development of Insomnia in patients with stroke, the authors report conducting post-hoc subgroup analyses to explore possible causes of heterogeneity among study results, indicating the potential effect modifiers considered and how they were defined:**  “But the heterogeneity was in a high level (I^2^: 93%). Due to the relatively small number of articles, data could not be removed to eliminate heterogeneity, and the source of heterogeneity was unclear.” |
|  | 13f | Describe any sensitivity analyses conducted to assess robustness of the synthesized results. | **In a review investigating the development of Insomnia in patients with stroke,** **the authors report conducting onel sensitivity analysis:**  “Meta-analysis of the prevalence of insomnia in stroke patients during follow-up.” |
| Reporting bias assessment | 14 | Describe any methods used to assess risk of bias due to missing results in a synthesis (arising from reporting biases). | **In a review investigating the development of Insomnia in patients with stroke, the authors report using funnel plots to assess study effects, noting that publication bias is one of several reasons for any asymmetry detected:**  “Ratio ratios (OR) and 95% confidence intervals (CI) were calculated for follow-up data. Statistical analyses were performed using Systematic Meta-Analysis software version 3.” |
| Certainty assessment | 15 | Describe any methods used to assess certainty (or confidence) in the body of evidence for an outcome. | **In a review investigating the development of Insomnia in patients with stroke, the authors report using the Critical Appraisal Tool for Prevalence Studies for assessing certainty in the body of evidence, stating how many reviewers performed assessments, the domains considered, and software used to perform assessments:**  “The methodological quality of the included studies was assessed using the Critical Appraisal Tool for Prevalence Studies [12-13]. If 2 researchers disagreed on the assessment of the quality of the literature, the decision was submitted to a third researcher (YJW).” |
| **RESULTS** | | |  |
| Study selection | 16a | Describe the results of the search and selection process, from the number of records identified in the search to the number of studies included in the review, ideally using a flow diagram. | **In a review investigating the development of Insomnia in patients with stroke, the authors report results of the search and selection process in text and in a flow diagram:**  “We got 1507 literatures from databases, of which 469 were duplicates and hence excluded. Further, we excluded 927 studies by the exclusion criteria. Overall, 111 studies were retained for the full-text evaluation, and finally 26 studies were included in the meta-analysis. (Fig.2).”  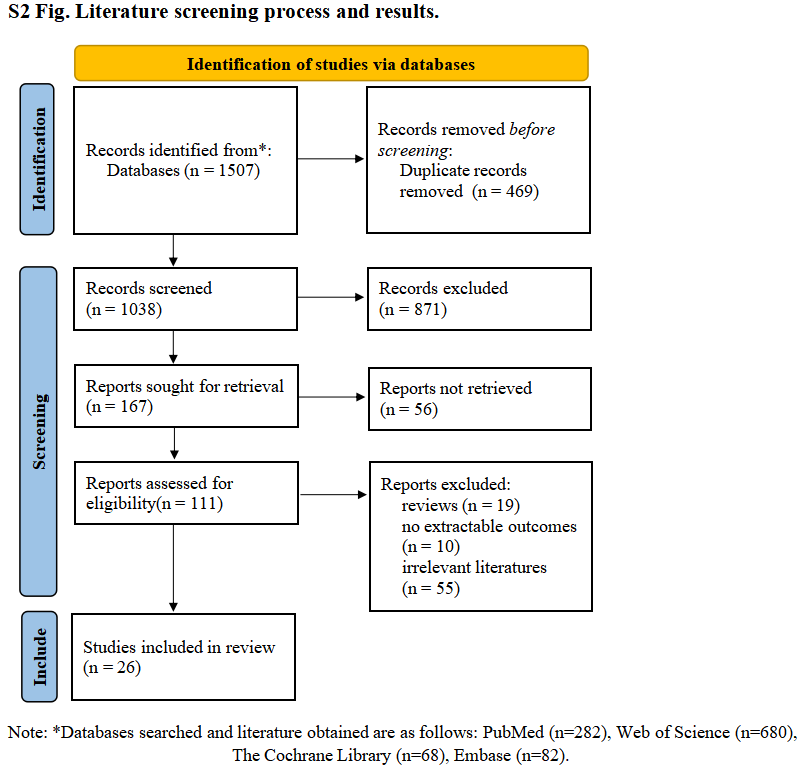 |
|  | 16b | Cite studies that might appear to meet the inclusion criteria, but which were excluded, and explain why they were excluded. | **In a review investigating the development of Insomnia in patients with stroke, the authors did not cite excluded studies.** |
| Study characteristics | 17 | Cite each included study and present its characteristics. | **In a review investigating the development of Insomnia in patients with stroke, the authors include a table presenting for each included study the citation, study design, country, sample size, number of years follow-up:**  “We got 1507 literatures from databases, of which 469 were duplicates and hence excluded. Further, we excluded 927 studies by the exclusion criteria. Overall, 111 studies were retained for the full-text evaluation, and finally 26 studies were included in the meta-analysis.  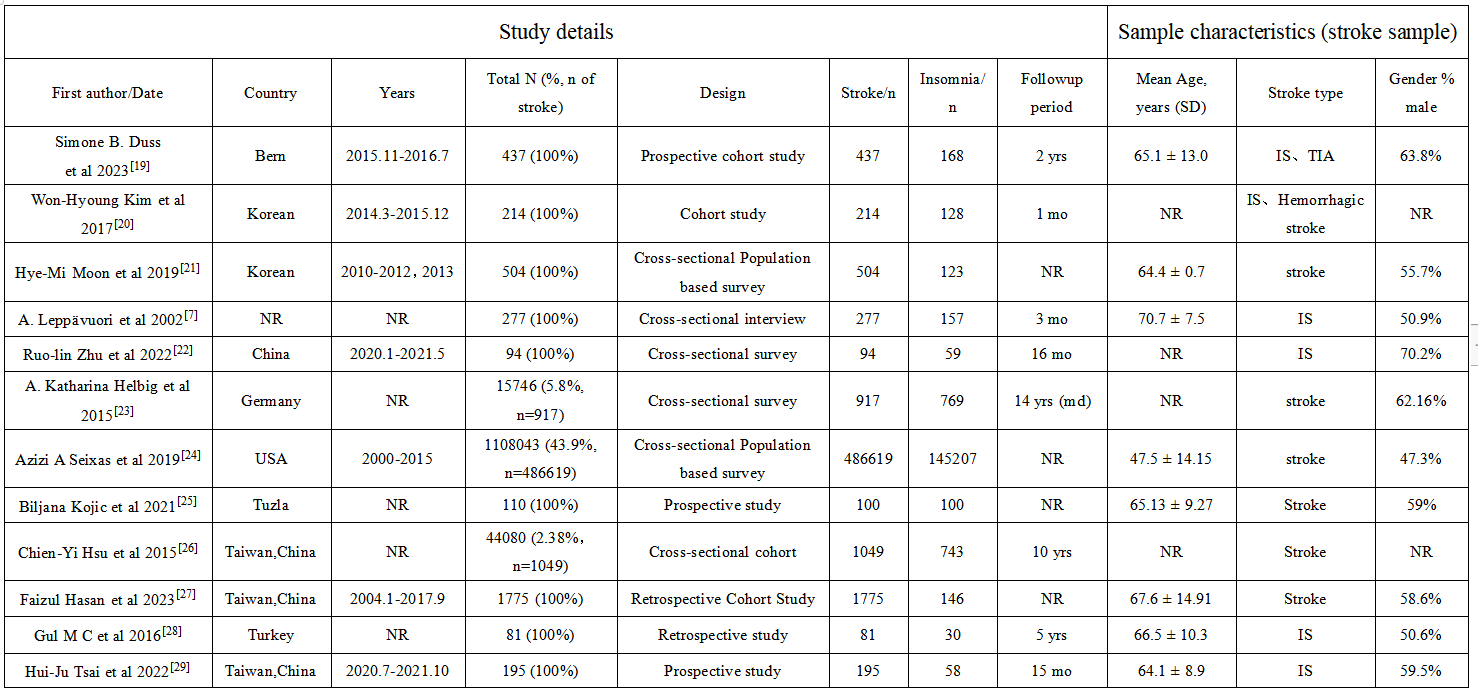  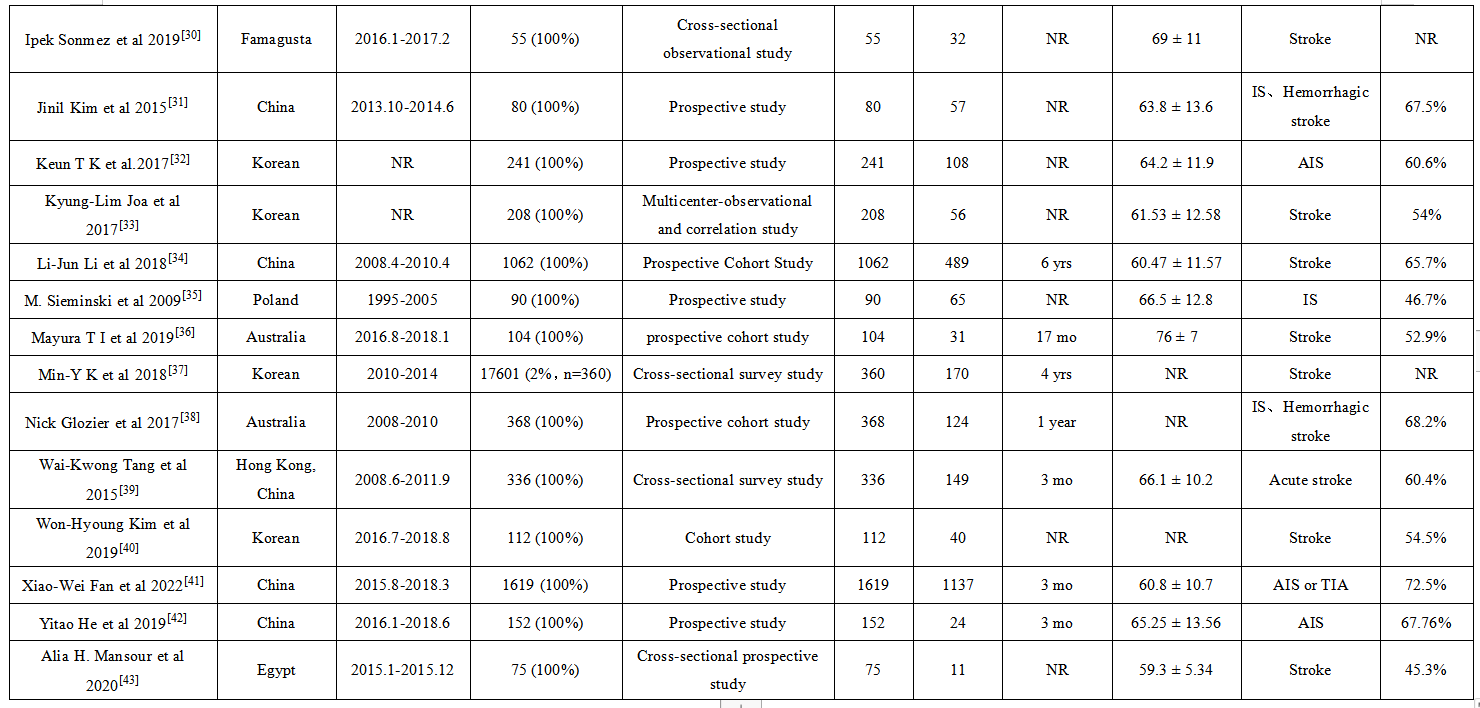 |
| Risk of bias in studies | 18 | Present assessments of risk of bias for each included study. | **In a review investigating the development of Insomnia in patients with stroke, the authors present a forest plot displaying risk of bias judgements for each study:**  “We used the random-effects model to pool prevalence of insomnia in patients with stroke. 150,181 patients with stroke developed insomnia during the follow-up and the pool prevalence was 46.98% (95% CI: 36.91–57.18). (Fig.3).” |
| Results of individual studies | 19 | For all outcomes, present, for each study: (a) summary statistics for each group (where appropriate) and (b) an effect estimate and its precision (e.g. confidence/credible interval), ideally using structured tables or plots. | **In a review investigating the development of Insomnia in patients with stroke, the authors present forest plots showing summary statistics for each group and effect estimates with confidence intervals for each study:**  “We used the random-effects model to pool prevalence of insomnia in patients with stroke. 150,181 patients with stroke developed insomnia during the follow-up and the pool prevalence was 46.98% (95% CI: 36.91–57.18).(Fig.3).” |
| Results of syntheses | 20a | For each synthesis, briefly summarise the characteristics and risk of bias among contributing studies. | **In a review investigating the development of Insomnia in patients with stroke, the authors summarise various characteristics and the risk of bias in studies investigating Insomnia in patients with Stroke:**  “(1) Twenty-six studies met the inclusion criteria for meta-analysis, with 1,193,659 participants, of which 497,124 were patients with stroke.  (2) The meta-analysis indicated that 150,181 patients with stroke developed insomnia during follow-up [46.98%, 95% confidence interval (CI): 36.91–57.18] and 1806 patients with ischemic stroke (IS) or transient ischemic attack (TIA) developed insomnia (47.21%, 95% CI: 34.26–60.36). Notably, 41.51% of patients with the prevalence of nonclassified stroke developed insomnia (95% CI: 28.86–54.75). The incidence of insomnia was significantly higher in patients with acute strokes than in patients with nonacute strokes (59.16% vs 44.07%, P < 0.0001).  (3) Similarly, the incidence of insomnia was significantly higher in the patients with stroke at a mean age of ≥65 than patients with stroke at a mean age of <65 years (47.18% vs 40.50%, P < 0.05). Fifteen studies reported the follow-up time. The incidence of insomnia was significantly higher in the follow-up for ≥3 years than follow-up for <3 years (58.06% vs 43.83%, P < 0.05). Twenty-one studies used the Insomnia Assessment Diagnostic Tool, and the rate of insomnia in patients with stroke was 49.31% (95% CI: 38.59–60.06). Five studies used self-reporting, that the rate of insomnia in patients with stroke was 37.58% (95% CI: 13.44–65.63).” |
|  | 20b | Present results of all statistical syntheses conducted. If meta-analysis was done, present for each the summary estimate and its precision (e.g. confidence/credible interval) and measures of statistical heterogeneity. If comparing groups, describe the direction of the effect. | **In a review investigating the development of Insomnia in patients with stroke, the authors report for a metaanalysis of the number of included studies and participants, summary estimate and its 95% confidence interval, the I^2^ measure of inconsistency:**  “We used the random-effects model to pool prevalence of insomnia in patients with stroke. 150,181 patients with stroke developed insomnia during the follow-up and the pool prevalence was 46.98% (95% CI: 36.91–57.18). (Fig.3).”  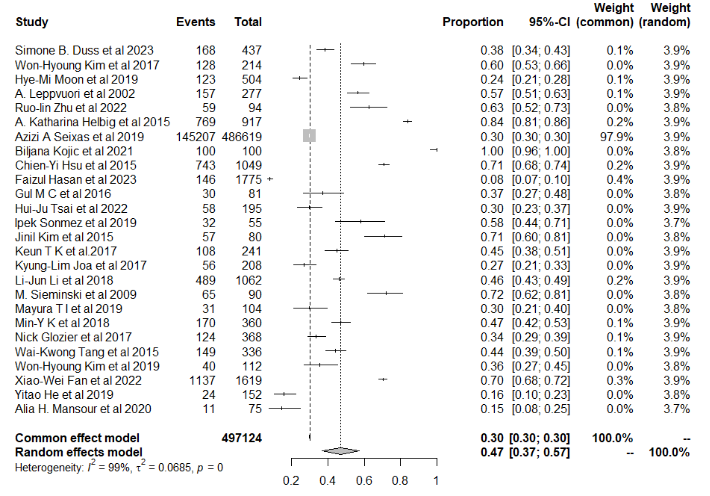 |
|  | 20c | Present results of all investigations of possible causes of heterogeneity among study results. | **In a review investigating the development of Insomnia in patients with stroke, the authors present results of several meta analyses, indicating for each the confidence interval, Due to the relatively small number of articles, data could not be removed to eliminate heterogeneity, and the source of heterogeneity was unclear.**  “We used the random-effects model to pool prevalence of insomnia in patients with stroke. 150,181 patients with stroke developed insomnia during the follow-up and the pool prevalence was 46.98% (95% CI: 36.91–57.18). (Fig.3).”  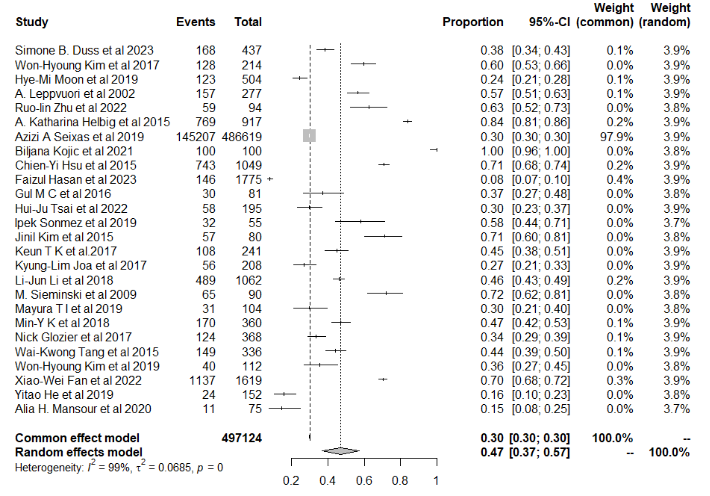 |
|  | 20d | Present results of all sensitivity analyses conducted to assess the robustness of the synthesized results. | **In a review investigating the development of Insomnia in patients with stroke,** **the authors report conducting onel sensitivity analysis:**  “Meta-analysis of the prevalence of insomnia in stroke patients during follow-up.” |
| Reporting biases | 21 | Present assessments of risk of bias due to missing results (arising from reporting biases) for each synthesis assessed. | **In a review investigating the development of Insomnia in patients with stroke, the authors did not report biaese.** |
| Certainty of evidence | 22 | Present assessments of certainty (or confidence) in the body of evidence for each outcome assessed. | **In a review investigating the development of Insomnia in patients with stroke, the authors did not report certainty of evidence.** |
| **DISCUSSION** | | |  |
| Discussion | 23a | Provide a general interpretation of the results in the context of other evidence. | **In a review investigating the development of Insomnia in patients with stroke, the authors compare their findings with those observed in other relevant reviews:**  “Our study found that the rate of insomnia after stroke (48.37%) was 1.27 times higher compared with the prevalence in the meta-analysis by Baylan et al. in 2019 (38.2%) [44]. It indicated that the prevalence of post-stroke insomnia continued to increase yearly, and insomnia had a significant negative impact on patients.” |
|  | 23b | Discuss any limitations of the evidence included in the review. | **In a review investigating the development of Insomnia in patients with stroke, the authors describe various limitations of the included studies:**  “This study had some limitations. First, the study quality was not an exclusion criterion, which might have contributed to the differences in the prevalence of insomnia after stroke. Studies used different tools for assessing and diagnosing insomnia, which might also have led to biased conclusions. Second, we did not study the treatment of patients with stroke and its effect on the development of insomnia.” |
|  | 23c | Discuss any limitations of the review processes used. | **In a review investigating the development of Insomnia in patients with stroke, the authors report several limitations of the review processes used:**  “This study had some limitations. First, the study quality was not an exclusion criterion, which might have contributed to the differences in the prevalence of insomnia after stroke. Studies used different tools for assessing and diagnosing insomnia, which might also have led to biased conclusions. Second, we did not study the treatment of patients with stroke and its effect on the development of insomnia.” |
|  | 23d | Discuss implications of the results for practice, policy, and future research. | **In a review investigating the development of Insomnia in patients with stroke, the authors discuss the implications for practice given the evidence of benefit observed:**  “Stroke may be a predisposing factor for insomnia. Insomnia is more likely to occur in acute-phase stroke, and the prevalence of insomnia increases with patient age and follow-up. Further, the rate of insomnia is higher in patients with stroke who use the Insomnia Assessment Diagnostic Tool.” |
| **OTHER INFORMATION** | | |  |
| Registration and protocol | 24a | Provide registration information for the review, including register name and registration number, or state that the review was not registered. | **In a review investigating the development of Insomnia in patients with stroke, the authors report that the review was registered, specifying the register name (PROSPERO) and registration number:**  “Registration number: CRD42023452419.” |
|  | 24b | Indicate where the review protocol can be accessed, or state that a protocol was not prepared. | **In a review investigating the development of Insomnia in patients with stroke, the authors report that the protocol for the review is published and provide a citation for it:**  “The review protocol was registered with the International Prospective Register of Systematic Reviews (PROSPERO) database (registration number: CRD42023452419).” |
|  | 24c | Describe and explain any amendments to information provided at registration or in the protocol. | **In a review investigating the development of Insomnia in patients with stroke, the authors describe and explain none amendments to information provided in the protocol.** |
| Support | 25 | Describe sources of financial or non-financial support for the review, and the role of the funders or sponsors in the review. | **In a review investigating the development of Insomnia in patients with stroke, the authors report receiving government agency funding to conduct the review, and describe the role of the funder and sponsor in the review:**  “Funding: This work was supported by Administration of Traditional Chinese medicine in guangxi, self-financing scientific research subject[grant numbers GXZYA20220072]; National Natural Science Foundation of  China [grant numbers 2022JJA141329]; Hospital scientific research project of the First Affiliated Hospital of Guangxi University of Traditional Chinese Medicine[grant numbers 2021QN008]; Guangxi University of Traditional Chinese Medicine research project[grant numbers 2022QN019].. juanjuan XIE provided intellectual input and supervision during the study process and made a substantial contribution to manuscript drafting.” |
| Competing interests | 26 | Declare any competing interests of review authors. | **In a review investigating the development of Insomnia in patients with stroke, the authors declare having no competing interests:**  “The authors declare that no conflict of interest.” |
| Availability of data, code and other materials | 27 | Report which of the following are publicly available and where they can be found: template data collection forms; data extracted from included studies; data used for all analyses; analytic code; any other materials used in the review. | **In a review investigating the development of Insomnia in patients with stroke, the authors report that the origin of each data will be uploaded in the future.** |
