# Supplementary material for: Development of insomnia in patients with stroke: A systematic review and meta-analysis
Source: PLoS One. 2024 Apr 10;19(4):e0297941. doi: 10.1371/journal.pone.0297941 (PMC11006172; doi:10.1371/journal.pone.0297941)
Supplement: S1 File — (PDF) [file pone.0297941.s003.pdf]

S2 Fig. Literature screening process and results.

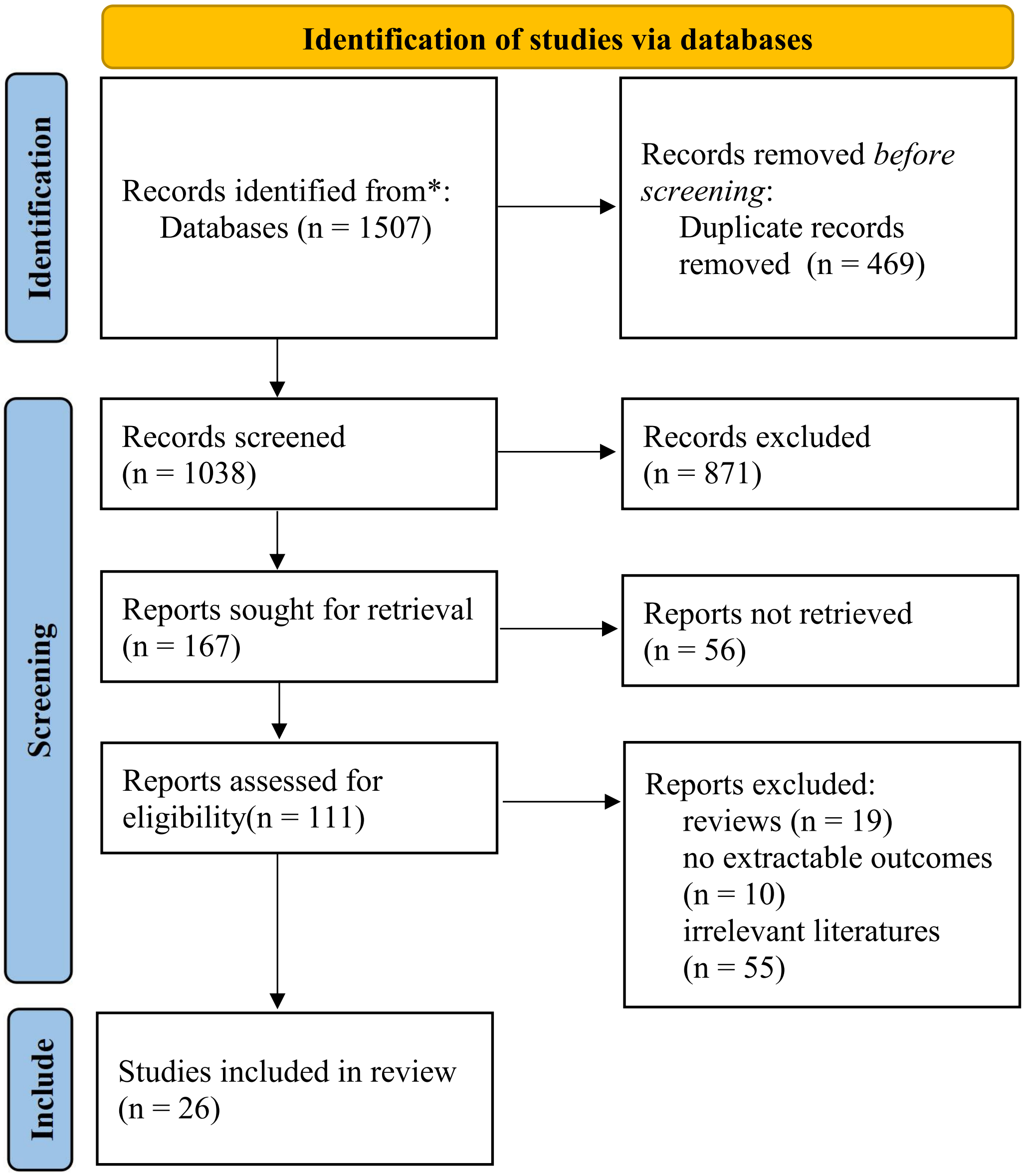

Note: *Databases searched and literature obtained are as follows: PubMed (n=282), Web of Science (n=680), The Cochrane Library (n=68), Embase (n=82).
